# CML20 integrates phytochrome and calcium signaling pathways to optimize seed germination under abiotic stress

**DOI:** 10.1038/s41467-026-77567-0

**Published:** 2026-09-04

**Authors:** Jing Kang, Jing Li, Xiaoyang Liu, Shuai Zhao, Shangwei Zhong, Hui Shi

**Affiliations:** 1 College of Life Sciences, Capital Normal University, Beijing, China; 2 Peking University Institute of Advanced Agricultural Sciences, Shandong Laboratory of Advanced Agricultural Sciences in Weifang, Weifang, China; 3 State Key Laboratory of Gene Function and Modulation Research, Peking-Tsinghua Center for Life Sciences, School of Life Sciences, Peking University, Beijing, China

**Keywords:** Light responses, Plant signalling, Abiotic

## Abstract

Plants integrate multiple environmental cues to decide the timing of seed germination. Light promotes germination primarily through photoreceptor phytochrome B (phyB), which eliminates the action of transcription factor PHYTOCHROME-INTERACTING FACTOR 1 (PIF1). Conversely, abiotic stresses typically elevate cytosolic Ca^2+^ and inhibit germination. However, the coordination of light and calcium signaling during germination under stress conditions remains poorly understood. Here, we demonstrate that salt and osmotic stresses rapidly increase cytosolic Ca^2+^ in imbibed seeds, and that the calcium sensor CALMODULIN-LIKE 20 (CML20) is crucial for germination tolerance to these stresses. CML20 directly interacts with PIF1 in a Ca^2+^-dependent manner, suppressing PIF1 self-association, oligomerization, and DNA-binding capacity. Moreover, phyB interacts with CML20 and PIF1 independently, promoting the assembly of the CML20-PIF1 complex and further reducing PIF1’s transcriptional activity. Genetic analyses reveal that PIF1 functions downstream of CML20, and that CML20 and phyB operate within the same pathway. Together, our findings define a phyB-CML20-PIF1 module, providing a rapid, reversible, and non-proteolytic mechanism to optimize seed germination under stress conditions.

## Abstract (web summary)

Plants integrate multiple environmental cues to time seed germination. Here, the authors show that the calcium-sensing CML20 protein links light and stress signals, enabling seeds to germinate under salt and drought conditions.

## Introduction

Seed germination represents a critical developmental transition in the plant life cycle, allowing sessile organisms to initiate growth in response to favorable environmental cues^1^. This process is tightly regulated by external signals including light, temperature, and abiotic stresses such as drought and salinity^2–5^. Light is a key promotive signal for germination, whereas abiotic stresses typically inhibit germination, serving as an adaptive strategy to delay seedling establishment under suboptimal conditions^3,5,6^. The molecular mechanisms by which seeds integrate these inputs to balance timely germination with stress resilience remain largely unknown.

Plants utilize a suite of photoreceptors to sense and respond to light, among which phytochrome B (phyB), a red/far-red light receptor, plays a central role in photo-reversible controlling seed germination^6–11^. PhyB is a dimeric holoprotein comprising an N-terminal photosensory module and a C-terminal output module^12,13^. It covalently binds the linear tetrapyrrole chromophore, phytochromobilin, enabling reversible photoconversion between the inactive red light-absorbing Pr form and the active far-red light-absorbing Pfr form^14,15^. In darkness, phyB predominantly localizes to the cytoplasm in the Pr form^12^. Upon red light exposure, it converts into the Pfr form, translocates into the nucleus, and forms photobodies via liquid-liquid phase separation, where it initiates downstream signaling events^12,16–18^. A key nuclear function of phyB is direct binding to basic helix-loop-helix (bHLH) transcription factors termed PHYTOCHROME INTERACTING FACTORS (PIFs), thereby modulating gene expression programs^19–21^.

Among distinct PIFs, PIF1 is a master repressor of seed germination in the dark and light-triggered inactivation of PIF1 is essential for initiating germination^11,22–24^. In darkness, PIF1 proteins are highly accumulated and suppress germination by regulating genes involved in hormone metabolism, cell wall remodeling, and transcriptional networks^25–31^. Light exposure promotes phyB-PIF1 interaction, leading to PIF1 phosphorylation, ubiquitination, and proteasomal degradation^25,32–37^. Beyond degradation, PIF1 activity is fine-tuned by cofactors that modulate its transcriptional output, ensuring precise control over germination^38–43^.

Drought and salinity stresses are global issues that severely affect crop production and distribution^44,45^. As major abiotic stresses, both salinity and drought significantly impede plant growth and reduce crop yields^44,45^. A common early response to these stresses is a rapid increase in cytosolic Ca^2+^ levels, which serves as a universal second messenger involved in various stress signaling pathways^46,47^. Plants interpret Ca^2+^ signals through a range of sensor systems, including calcium-dependent protein kinases (CPKs/CDPKs), the calcineurin B-like protein/CIPK network, and calmodulins (CaMs) along with plant-specific calmodulin-like proteins (CMLs)^48,49^. CaM is a small acidic protein composed of four EF-hand Ca^2+^-binding motifs, lacking any catalytic domains. Ca^2+^ binding induces conformational changes that enable selective interactions with target proteins^50–53^. Plants have expanded this module through a large family of CMLs. The Arabidopsis genome encodes approximately 50 CMLs, each with distinct Ca^2+^ affinities, expression patterns, and partner specificities, reflecting the complexity of Ca^2+^ decoding mechanisms^54–57^. Despite this expansion, the physiological roles and direct targets of most CMLs remain unknown.

In this study, we identify a Ca^2+^-sensor-based mechanism that integrates light and stress signals during seed germination. We show that CML20 connects light and stress-encoded Ca^2+^ signals by directly binding and suppressing PIF1 in a Ca^2+^-dependent manner. Furthermore, phyB acts as a scaffold to promote the formation of CML20-PIF1 complex, thereby dampening PIF1’s activity and alleviating the inhibition of seed germination under salinity and drought stresses. This phyB-CML20-PIF1 module provides a mechanistic framework for integrating light and stress cues at the molecular level to optimize seed germination.

## Results

### PhyB is essential for light-promoted germination tolerance to salt and osmotic stress

**Fig. 1:**
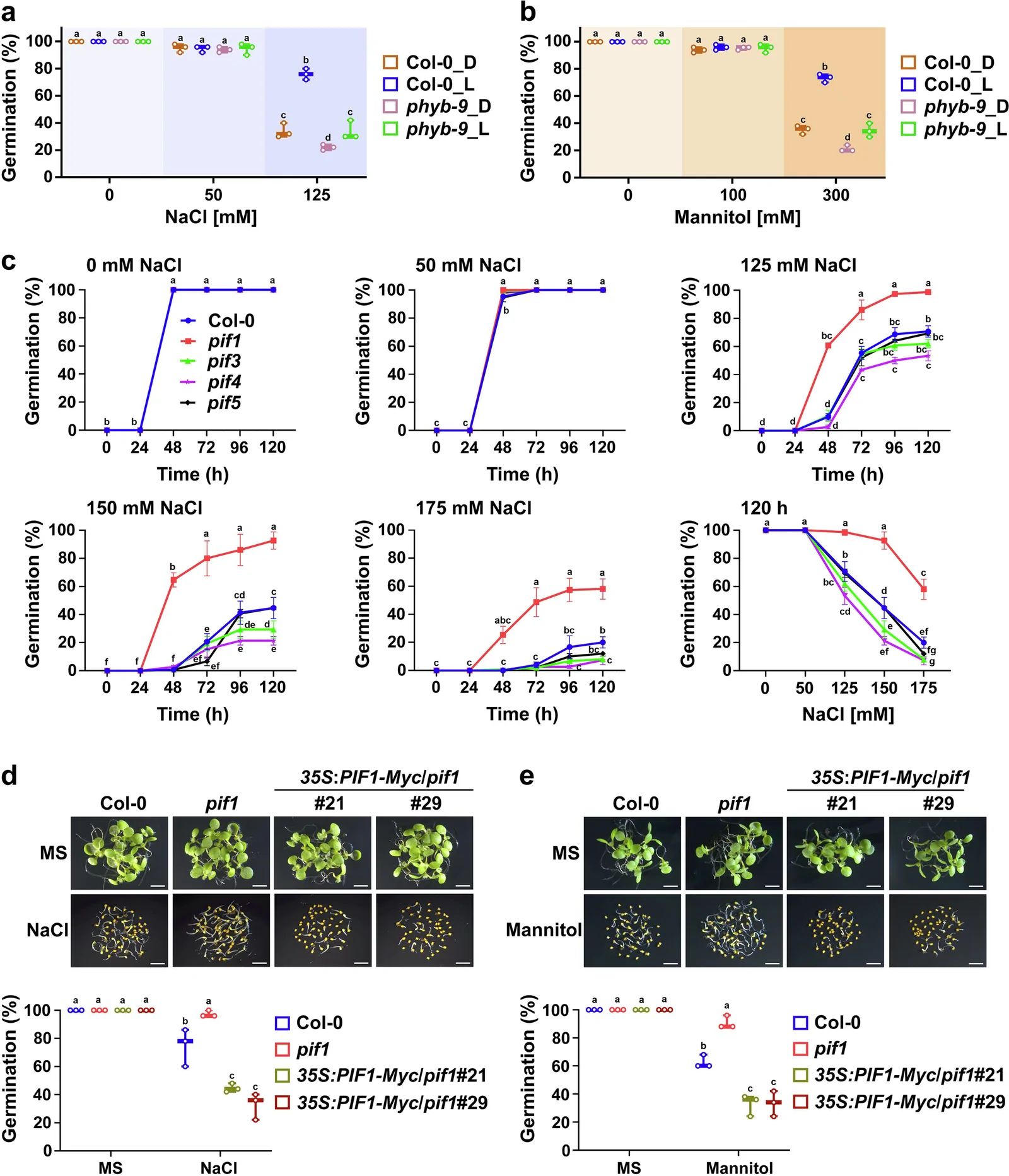
PhyB and PIF1 antagonistically regulate seed germination under salt and osmotic stress. **a, b** Germination rates of Col-0 and *phyb-9* seeds on MS medium supplemented with the indicated concentrations of NaCl (**a**) or mannitol (**b**) under continuous white light (Light) or darkness (Dark). Seeds were sown on MS medium containing NaCl (**a**) or mannitol (**b**), stratified at 4 °C for 3 d in the dark, then exposed to white light for 8 h to induce germination, and subsequently incubated in either continuous white light (Light, L) or darkness (Dark, D) for 5 days. *n* = 3 biological replicates. Two-way ANOVA followed by Tukey’s multiple comparisons test; different letters indicate significant differences (*p* < 0.05). **c** Time-course germination of Col-0, *pif1*, *pif3*, *pif4*, and *pif5* seeds on MS medium with the indicated NaCl concentrations under continuous white light. Seeds were stratified at 4 °C for 3 d in the dark, and incubated in continuous white light. Germination was recorded every 24 h at the indicated time points. Data are means ± SD; *n* = 3 biological replicates. Two-way ANOVA followed by Tukey’s multiple comparisons test; different letters indicate significant differences (*p* < 0.05). **d**, **e** Representative images (top) and germination rates (bottom) of Col-0, *pif1*, and two independent *35S:PIF1-Myc*/*pif1* lines under salt (**d**) or osmotic (**e**) stress. Seeds were sown on MS medium containing 125 mM NaCl (**d**) or 300 mM mannitol (**e**), stratified at 4 °C for 3 days in the dark, and incubated in continuous white light for 5 days. Scale bar, 2 mm. *n* = 3 biological replicates. Two-way ANOVA followed by Tukey’s multiple comparisons test; different letters indicate significant differences (*p* < 0.05). Source data and the exact *p*-values are provided in the Source Data file.

Phytochromes have been implicated in promoting salt-stress tolerance of Arabidopsis seedlings^58,59^. As phyB is the dominant photoreceptor controlling light-dependent germination, we investigated its role in stress tolerance during seed germination. Wild‑type (WT) and *phyb‑9* mutant seeds were incubated on MS medium supplemented with NaCl (salt stress) or mannitol (osmotic stress) in darkness (Dark, D) or continuous white light (Light, L). On MS control medium, both WT and *phyb-9* seeds germinated fully, confirming seed viability (Fig. 1a). Addition of 50 mM NaCl did not significantly affect germination of either WT or *phyb-9* (Fig. 1a). However, under 125 mM NaCl, WT germination reached ~35% in Dark and ~75% in Light (Fig. 1a). In contrast, *phyb-9* germinated at only ~35% in Light, comparable to WT in Dark, and at ~20% in Dark (Fig. 1a). Similar patterns were observed for mannitol treatment (Fig. 1b). The modest light response in *phyb-9* likely reflects compensation by other photoreceptors (e.g., phyA). These results indicate that phyB is required for light-promoted germination under salt and osmotic stress.

To precisely assess the role of phyB, we performed phyB‑dependent seed germination assay under salt or osmotic stress. Seeds were treated with far‑red light (phyB‑OFF) or far‑red followed by red light (phyB‑ON) before being incubated in darkness. Under phyB‑OFF conditions, WT seeds did not germinate even on control medium (Suppl. Fig. 1a). Under phyB‑ON conditions, WT germination exceeded 90% on control medium and declined to ~40% with 50 mM NaCl and ~20% with 125 mM NaCl (Suppl. Fig. 1a). The *phyb‑9* mutant failed to germinate under both phyB-ON and phyB-OFF conditions, similar to WT under phyB‑OFF conditions (Suppl. Fig. 1a). Osmotic stress produced similar patterns (Suppl. Fig. 1b). Collectively, these data demonstrate the essential role of phyB in light‑promoted tolerance to salt and osmotic stresses during seed germination.

### PIF1 negatively regulates seed germination under salt and osmotic stress

Given that PIF transcription factors act downstream of phyB, we examined whether individual PIF (PIF1, PIF3, PIF4 or PIF5) contribute to seed germination under stress using single *pif* mutants. On MS medium without or with 50 mM NaCl, all genotypes germinated completely within 48 h with no discernible differences (Fig. 1c). However, the *pif1* seeds were less sensitive to NaCl at higher NaCl. On MS medium supplemented with 125, 150 and 175 mM NaCl, WT germination was strongly suppressed, whereas *pif1* mutant germinated earlier and achieved higher final germination rates than WT (Fig. 1c). In contrast, the *pif4* mutant showed a modest difference in germination, whereas *pif3* and *pif5* were indistinguishable from WT across all NaCl concentrations tested (Fig. 1c). On mannitol-supplemented medium, *pif1* mutant germinated markedly faster and reached higher final germination than WT, while *pif3*, *pif4*, and *pif5* displayed hypersensitivity to mannitol treatment (Suppl. Fig. 2a). These observations suggest that PIF1 negatively regulates seed germination under salt and osmotic stress.

To further validate PIF1’s role, we generated transgenic plants overexpressing *PIF1*-*Myc* under the constitutive *35S* promoter in the *pif1* background (*35S*:*PIF1-Myc*/*pif1*). RT-qPCR confirmed elevated *PIF1* gene expression in two independent lines compared to WT (Suppl. Fig. 2b). WT, *pif1* mutant and *35S*:*PIF1-Myc*/*pif1* seeds fully germinated on MS medium (Fig. 1d, Suppl. Fig. 2c). Under 125 mM NaCl, in contrast to the insensitivity of *pif1* mutant to salt stress, both overexpression lines were hypersensitive, exhibiting delayed and achieving only 30–40% final germination compared with over 60% of WT (Fig. 1d, Suppl. Fig. 2c). Similar hypersensitivity was observed for *35S*:*PIF1-Myc*/*pif1* seeds on mannitol-supplemented medium (Fig. 1e, Suppl. Fig. 2d). Taken together, these results suggest that PIF1 is a key repressor of germination under salt and osmotic stress.

### CML20 promotes seed germination under salt and osmotic stress

**Fig. 2:**
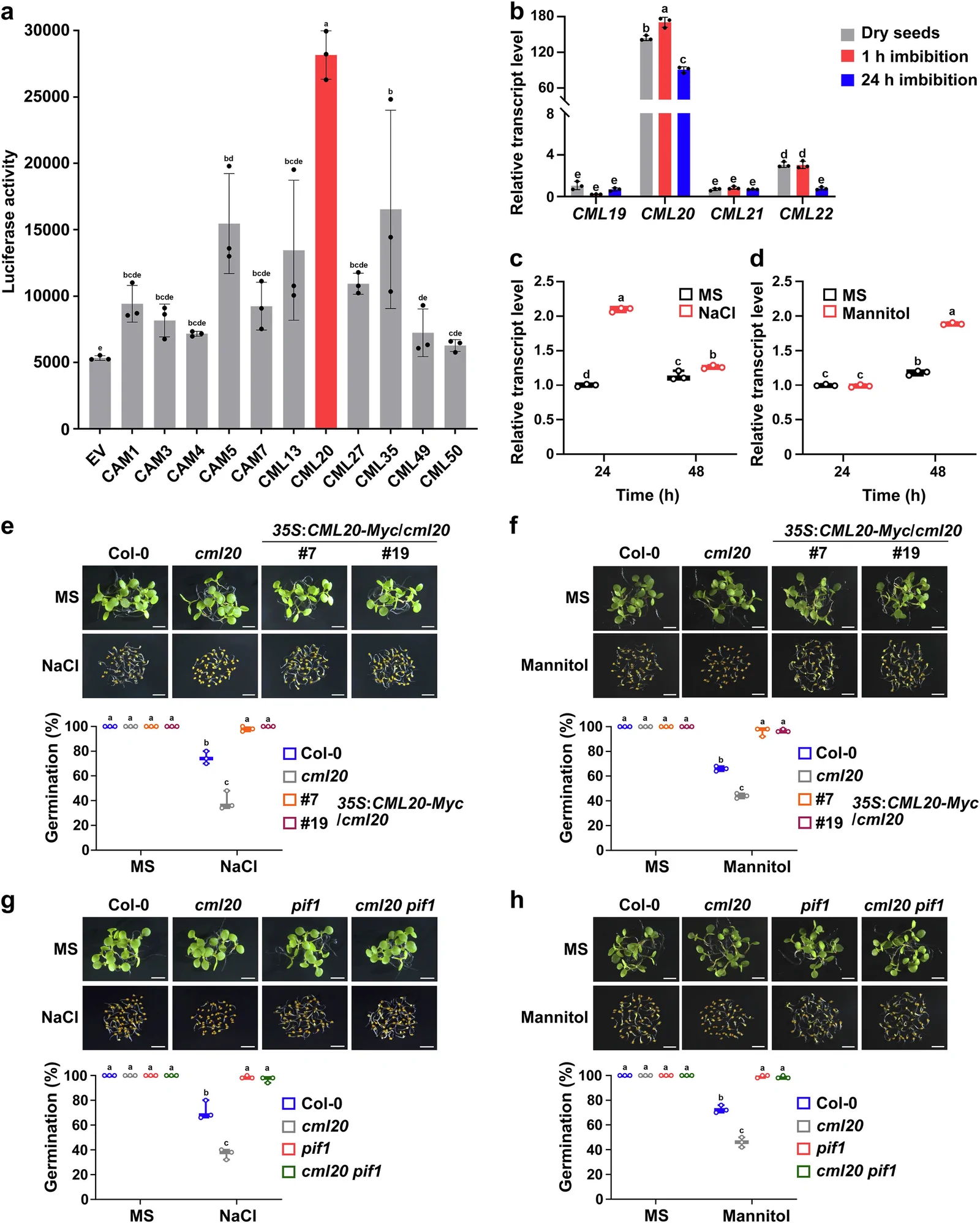
CML20 acts genetically upstream of PIF1 to promotes seed germination under salt and osmotic stress. **a** LCI assays showing the interactions between PIF1 and CAM/CML proteins in vivo. PIF1 was fused to cLuc, and CAM/CML protein was fused to nLuc, respectively. Data are means ± SD; *n* = 3 biological replicates. One-way ANOVA followed by Tukey’s multiple comparisons test; different letters indicate significant differences (*p* < 0.05). **b** RT-qPCR analysis of subgroup V *CML* transcripts in mature dry and imbibed seeds. Col-0 seeds were stratified at 4 °C in darkness and were imbibed in continuous white light for 1 h or 24 h. *ACTIN2* served as the internal control. Data are means ± SD; *n* = 3 technical replicates. Two-way ANOVA followed by Tukey’s multiple comparisons test; different letters indicate significant differences (*p* < 0.05). **c**, **d** RT-qPCR analysis of *CML20* expression in imbibed seeds treated with NaCl (**c**) or mannitol (**d**). Col-0 seeds were stratified at 4 °C in darkness and soaked in liquid MS without or with 125 mM NaCl (**c**) or 300 mM mannitol (**d**) for 24 h or 48 h. *UBQ5* served as the internal control. *n* = 3 technical replicates. Two-way ANOVA followed by Tukey’s multiple comparisons test; different letters indicate significant differences (*p* < 0.05). **e–h** Representative images (top) and germination rates (bottom) of the indicated genotypes under salt (**e**, **g**) or osmotic (**f**, **h**) stress. Genotypes for each panel are as follows: (**e**, **f**) Col-0, *cml20*, and two independent *35S:CML20-Myc*/*cml20* lines; (**g**, **h**) Col-0, *cml20*, *pif1* and *cml20 pif1*. Seeds were sown on MS medium containing 125 mM NaCl (**e**, **g**) or 300 mM mannitol (**f**, **h**), stratified at 4 °C for 3 d in the dark, and incubated in continuous white light for 5 days. Scale bar, 2 mm. *n* = 3 biological replicates. Two-way ANOVA followed by Tukey’s multiple comparisons test; different letters indicate significant differences (*p* < 0.05). Source data and the exact *p*-values are provided in the Source Data file.

Distinct stress cues generate characteristic Ca^2+^ signatures that are decoded by specific calcium sensors^49^. Mining published Arabidopsis transcriptomes revealed that several *CaM*/*CML* genes are enriched in seeds^55^ (Suppl. Fig. 3a). To identify seed-expressed Ca^2+^ sensors that might interface with PIF1, we performed a luciferase complementation imaging (LCI) screen for PIF1 interactions across these CaM/CMLs. CALMODULIN-LIKE 20 (CML20) fused to nLuc generated the strongest reconstituted luminescence with cLuc-PIF1, indicating CML20 as a robust PIF1 interactor (Fig. 2a).

Phylogenetically, CML20 belongs to subclade V of the CML family, which includes four paralogs (CML19/20/21/22)^55^. Sequence alignment revealed that CML19, CML20, CML21, and CML22 all contain four EF-hand domains responsible for Ca^2+^ binding (Suppl. Fig. 3b). CML20 shares 64.74% sequence identity with CML19 but only 15.31% and 16.81% identity with CML21 and CML22, respectively (Suppl. Fig. 3b). AlphaFold predictions indicated similar topological structures for CML19 and CML20, which differ from those of CML21 and CML22 (Suppl. Fig. 3c). RT-qPCR analysis revealed that the transcript level of *CML20* was substantially higher than those of its paralogs *CML19*, *CML21*, and *CML22* in both dry and imbibed seeds (Fig. 2b). Furthermore, *CML20* transcripts were induced by NaCl or mannitol treatment in imbibed seeds (Fig. 2c, d), indicating that CML20 is responsive to salt and osmotic stress.

To test CML20 function genetically, we analyzed two T-DNA insertion mutants, *cml20* (SALK_079974) and *cml20-2* (CS925331) (Suppl. Fig. 4a). RT-qPCR showed that *CML20* transcript levels are markedly reduced in both *cml20* and *cml20-2* mutants (Suppl. Fig. 4b). In seed germination assays, WT, *cml20*, and *cml20-2* seeds all germinated fully on MS control medium (Suppl. Fig. 4c). Under 125 mM NaCl or 300 mM mannitol, WT germination was reduced, whereas *cml20* and *cml20-2* exhibited marked hypersensitivity, with significantly lower germination frequencies compared with WT (Suppl. Fig. 4c). These results indicate that CML20 positively regulates seed germination under salt and osmotic stress.

To further confirm that the observed phenotypes are specifically attributable to loss of *CML20* function, we generated transgenic lines expressing *Myc*-tagged *CML20* from the *35S* promoter in the *cml20* background (*35S*:*CML20-Myc*/*cml20*). RT-qPCR confirmed robust overexpression in two independent *35S*:*CML20-Myc*/*cml20* transgenic lines (Suppl. Fig. 5a). In seed germination assays, WT, *cml20*, and *35S*:*CML20-Myc*/*cml20* seeds all fully germinated on MS control medium (Fig. 2e, Suppl. Fig. 5b). Under 125 mM NaCl, WT germination was delayed and reduced to a final germination percentage of ~70%, whereas *cml20* was less than 40% final germination (Fig. 2e, Suppl. Fig. 5b). The *35S*:*CML20-Myc*/*cml20* lines fully rescued the *cml20* defect and, notably, exhibited even higher germination frequencies than WT under salt stress (Fig. 2e, Suppl. Fig. 5b). Similar results were observed under mannitol-induced osmotic stress: *cml20* seeds were hypersensitive, while *35S*:*CML20-Myc*/*cml20* seeds were more tolerant than WT (Fig. 2f, Suppl. Fig. 5c). Taken together, these results demonstrate that CML20 is a positive regulator of seed germination under salt and osmotic stress.

### CML20 acts genetically upstream of PIF1

Given the roles of PIF1 and CML20 in regulating seed germination under stress, we analyzed their genetic relationship. We generated the *cml20 pif1* double mutant and compared its germination with WT, *cml20*, and *pif1* single mutant seeds. All genotypes germinated completely on MS control medium, indicating comparable seed viability (Fig. 2g, h, Suppl. Fig. 6). At mild stress (50 mM NaCl or 100 mM mannitol), *cml20* already showed significant hypersensitivity, whereas *pif1* and *cml20 pif1* germinated similarly to WT (Suppl. Fig. 6). At higher stress levels (125–175 mM NaCl or 200–400 mM mannitol), germination decreased progressively in all genotypes, but *pif1* consistently germinated faster and achieved higher rates than WT, while *cml20* exhibited reduced germination (Fig. 2g, h, Suppl. Fig. 6). Notably, the *cml20 pif1* double mutant resembled *pif1* under both stresses and fully suppressed the *cml20* defective germination (Fig. 2g, h, Suppl. Fig. 6). These data demonstrate that CML20 functions upstream of PIF1 in mediating germination tolerance across a wide range of salt and osmotic stress conditions.

### CML20 interacts with PIF1 in vitro and in vivo

**Fig. 3:**
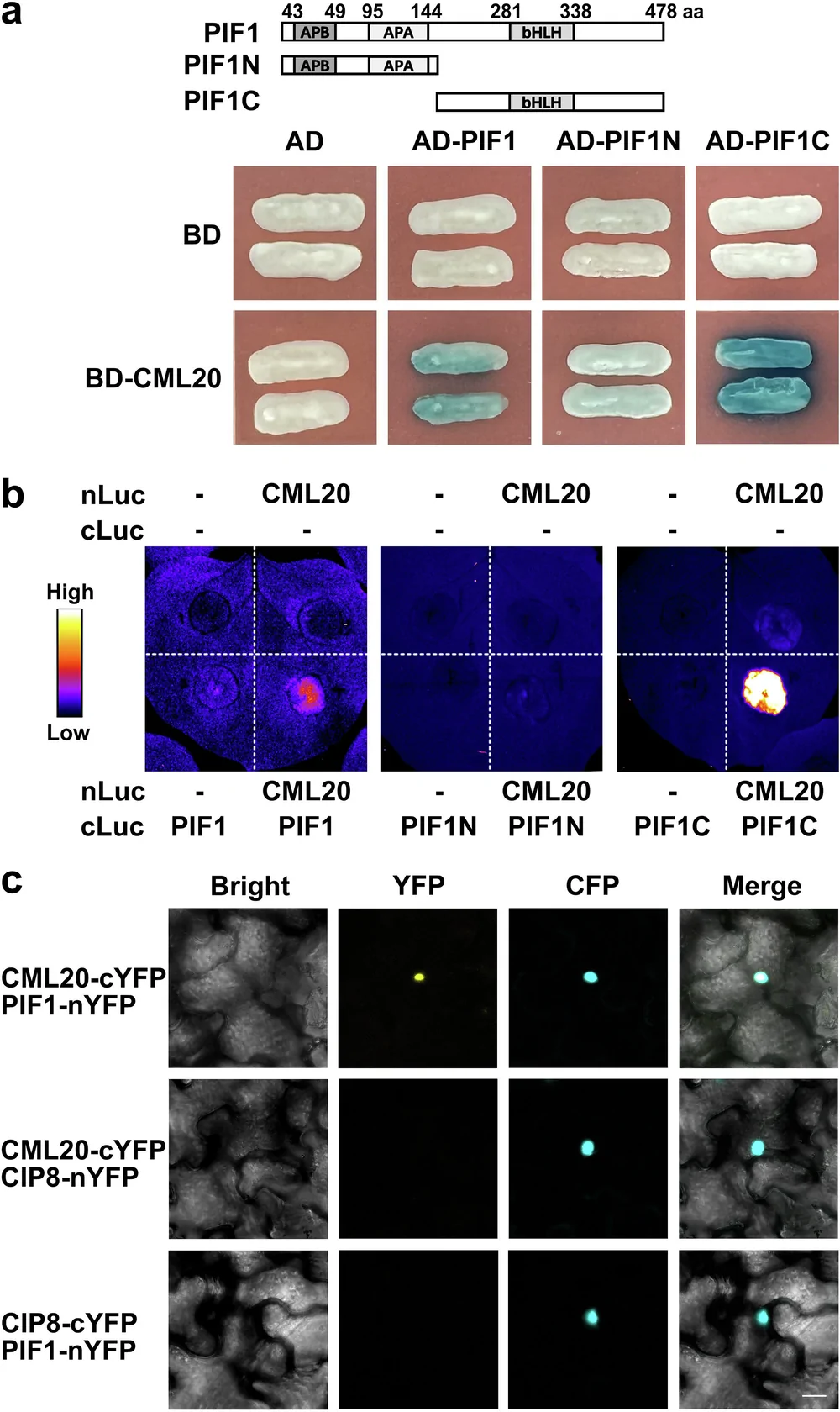
CML20 interacts with PIF1 in vitro and in vivo. **a** Yeast two-hybrid assay showing interactions between CML20 and full-length PIF1, the PIF1 N-terminal fragment (PIF1N), or the C-terminal fragment (PIF1C). Top diagram depicts the PIF1 fragments fused to AD; empty vectors serve as negative controls. **b** LCI assays showing CML20-PIF1 interactions in tobacco leaves. PIF1 fragments in (**a**) were fused to cLuc, and full-length CML20 was fused to nLuc. Empty vectors are negative controls. **c** BiFC assays showing interaction between CML20 and PIF1 in the nucleus of tobacco epidermal cells. CIP8 serves as a nuclear-localized negative control. H2A-CFP marks nuclei. Scale bar = 20 μm.

To investigate the regulatory mechanism of CML20 on PIF1, we first examined the protein-protein interactions between PIF1 and CML20. Yeast two-hybrid assays confirmed that PIF1 physically interacts with CML20 (Fig. 3a). Using PIF1 truncations, including the N-terminal region (PIF1N, containing the APA and APB motifs) and the C-terminal region (PIF1C, which contains the bHLH domain), we mapped the interacting domain. Our results showed that CML20 interacts strongly with PIF1C but not with PIF1N (Fig. 3a).

To further validate this interaction *in planta*, we conducted LCI assay in tobacco leaves. Full-length CML20 fused to nLuc produced strong luminescence when co-expressed with cLuc fusions of either full-length PIF1 or PIF1C, but not with PIF1N (Fig. 3b). These findings indicate that PIF1 interacts with CML20 through its C-terminus.

Bimolecular fluorescence complementation (BiFC) further confirmed this interaction and revealed its subcellular localization. Co-expression of CML20-cYFP with PIF1-nYFP resulted in strong nuclear YFP fluorescence in the epidermal cells of tobacco leaves (Fig. 3c). In contrast, no fluorescence was observed when either fusion was paired with the nuclear protein CIP8, which served as a negative control (Fig. 3c). This demonstrates the specificity of the CML20-PIF1 interaction in the nucleus of plant cells.

### PIF1 interacts with CML20 in a Ca^2+^-dependent manner

**Fig. 4:**
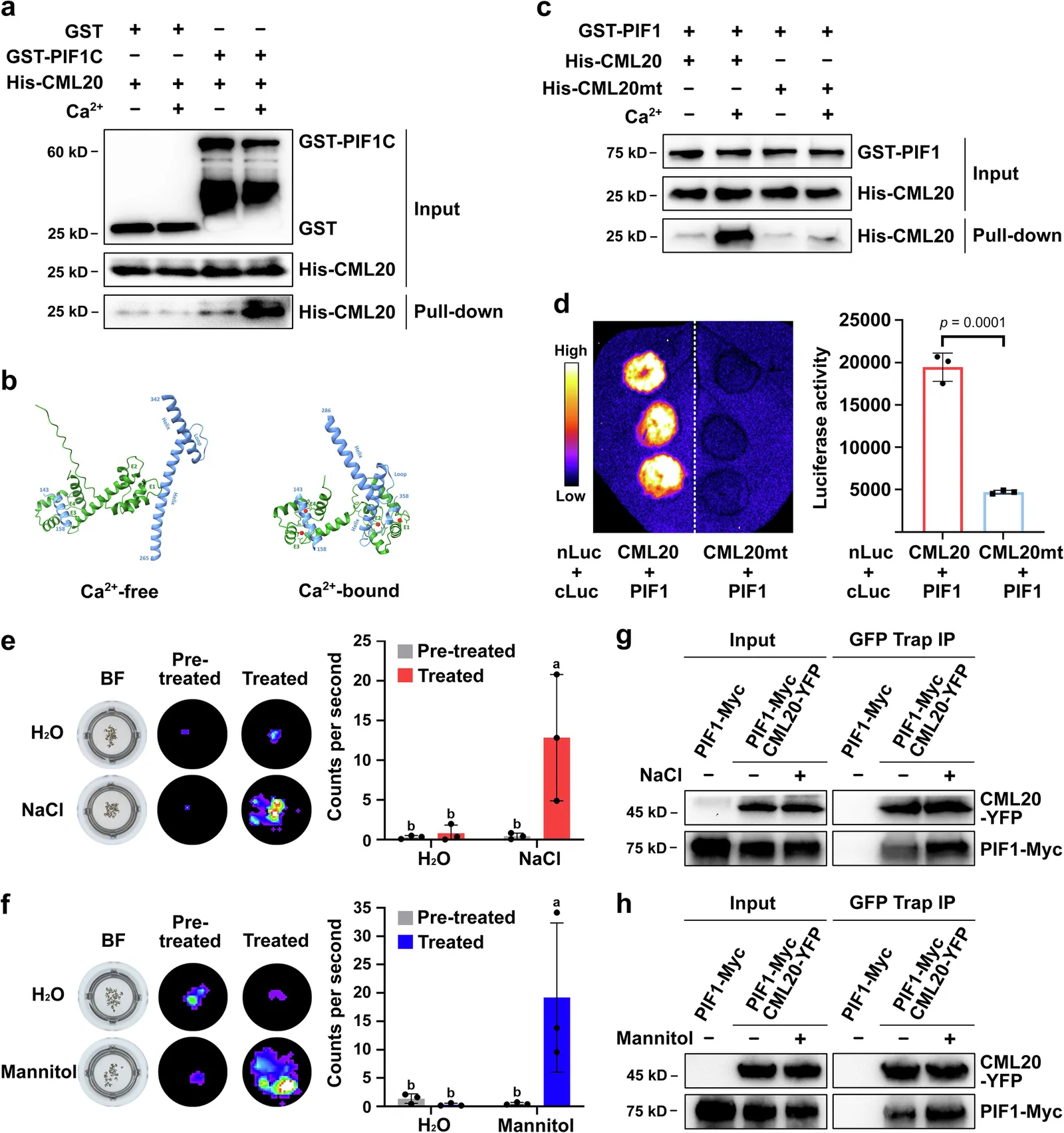
PIF1 interacts with CML20 in a Ca^2+^-dependent manner. **a** In vitro pull-down assays indicating direct Ca^2+^-dependent interactions between CML20 and PIF1C. Purified His-CML20 was incubated with GST-PIF1C or GST alone (negative control) in the presence (+) or absence (−) of Ca^2+^. Anti-GST nanobody agarose beads were used to pull down GST-PIF1C and associated His-CML20. **b** AlphaFold-predicted structures of CML20-PIF1 complexes without (left) and with (right) Ca^2+^. PIF1 (blue), CML20 (green), Ca^2+^ (red). Numbers denote PIF1 residue positions. E1–E4 indicate EF-hand motifs of CML20. **c** In vitro pull-down assays showing interactions between GST-PIF1 and His-CML20 or Ca^2+^-binding-deficient His-CML20mt (E83A/E156A) in the presence (+) or absence (−) of Ca^2+^. Anti-GST nanobody agarose beads were used to pull down GST-PIF1 and associated His-CML20 or His-CML20mt. **d** LCI assays showing Ca^2+^-dependent CML20-PIF1 interaction in vivo. Full-length PIF1 was fused to cLuc, and full-length CML20 or Ca^2+^-binding deficient version CML20mt was fused to nLuc. Data are means ± SD; *n* = 3 biological replicates. The *p*-value was determined by using a two‑sided Student’s *t*-test. **e**, **f** Representative aequorin bioluminescence images (left) and quantitative analysis (right) of cytosolic Ca^2+^ changes in AEQ‑expressing imbibed seeds following treatment with 125 mM NaCl (**e**) or 660 mM mannitol (**f**). Images were captured immediately before (Pre‑treated) and after (Treated) stress application. Luminescence intensity is presented as counts per second (CPS). Data are means ± SD; *n* = 3 biological replicates. Two-way ANOVA followed by Tukey’s multiple comparisons test; different letters indicate significant differences (*p* < 0.05). **g, h** Co-IP assay showing the association of CML20 and PIF1 in imbibed seeds in response to stresses. Seeds of *35S*:*PIF1-Myc* (PIF1-Myc) and *35S*:*PIF1-Myc*/*35S*:*CML20-YFP* (PIF1-Myc/CML20-YFP) were imbibed in the dark for 24 h and then treated with 125 mM NaCl (**g**) or 660 mM mannitol (**h**) for 1 h. Immunoprecipitation was performed using anti-GFP nanobody agarose beads. Source data and the exact *p*-values are provided in the Source Data file.

As a Ca^2+^-binding sensor, CML20 undergoes conformational changes upon Ca^2+^ binding^60^. To test whether Ca^2+^ modulates the CML20-PIF1 interaction, we performed in vitro pull-down assays under Ca^2+^-depleted and Ca^2+^-supplemented conditions. Strikingly, GST-PIF1C robustly pulled down His-CML20 only in the presence of Ca^2+^, whereas binding was negligible in the absence of Ca^2+^ and with GST alone (Fig. 4a), indicating the Ca^2+^-dependence of CML20-PIF1 interaction. Structural modeling using AlphaFold predicted that CML20 adopts two calmodulin-like globular domains with four EF-hands, and that Ca^2+^ binding switches CML20 from a closed to an open state, exposing hydrophobic surfaces likely used for PIF1 engagement (Fig. 4b).

The final glutamate in each EF-hand motif is conserved in CaM/CML proteins and crucial for Ca^2+^-dependent binding^55^. To further test whether Ca^2+^ coordination by EF-hands is necessary for CML20-PIF1 interaction, we substituted the conserved terminal glutamate to alanine in two EF-hand loops (E83A and E156A; referred to as CML20mt), a change shown to impair Ca^2+^ binding. In pull-down assays, interactions between GST-PIF1 and His-CML20 were induced by Ca^2+^ (Fig. 4c). However, the Ca^2+^-induced binding of GST-PIF1 to His-CML20 was strongly reduced for CML20mt compared to wild-type CML20 (Fig. 4c). Consistently, LCI results showed robust signal for PIF1 with CML20 but a significantly reduced signal with CML20mt (Fig. 4d). Together, these results demonstrate that the PIF1-CML20 association is Ca^2+^ dependent.

### Salt and osmotic stresses elevate cytosolic Ca^2+^ and enhance the PIF1-CML20 interaction in imbibed seeds

To determine whether stress conditions affect the CML20-PIF1 interaction in vivo, we first monitored cytosolic Ca^2+^ dynamics using transgenic plants expressing the Ca^2+^ sensitive luminescent reporter aequorin (AEQ)^46,61^. AEQ‑based imaging showed that treatment with NaCl or mannitol rapidly triggers a pronounced increase of cytosolic Ca^2+^ in imbibed seeds (Fig. 4e, f), confirming that salt and osmotic stresses elicit Ca^2+^ signals during seed imbibition.

We then performed co‑immunoprecipitation (co-IP) assays using protein extracts from seeds imbibed under NaCl or mannitol treatment. PIF1-Myc was specifically co-precipitated by GFP-Trap in samples co-expressing CML20-YFP (*35S*:*PIF1-Myc*/*35S*:*CML20-YFP*), but not in controls lacking CML20-YFP (*35S*:*PIF1-Myc*) (Fig. 4g, h). Notably, both NaCl and mannitol treatments increased the amount of PIF1‑Myc co‑precipitated with CML20‑YFP compared with the untreated control (Fig. 4g, h). To examine whether stress conditions alter the subcellular distribution of CML20, we performed nuclear-cytoplasmic fractionation followed by Western blotting using *35S:CML20-YFP/cml20* and *35S:CML20-Myc/cml20* transgenic seeds. Both CML20-YFP and CML20-Myc were detected in the nuclear and cytoplasmic fractions under all conditions, and stress treatment did not cause any obvious redistribution relative to the untreated control (Suppl. Fig. 7). These results demonstrate that CML20 does not undergo stress-induced subcellular relocalization. Collectively, these results demonstrate that salt and mannitol stresses trigger Ca^2+^ signals in imbibed seeds, which in turn enhance the interaction between CML20 and PIF1.

### CML20 inhibits PIF1 self-association and oligomerization

**Fig. 5:**
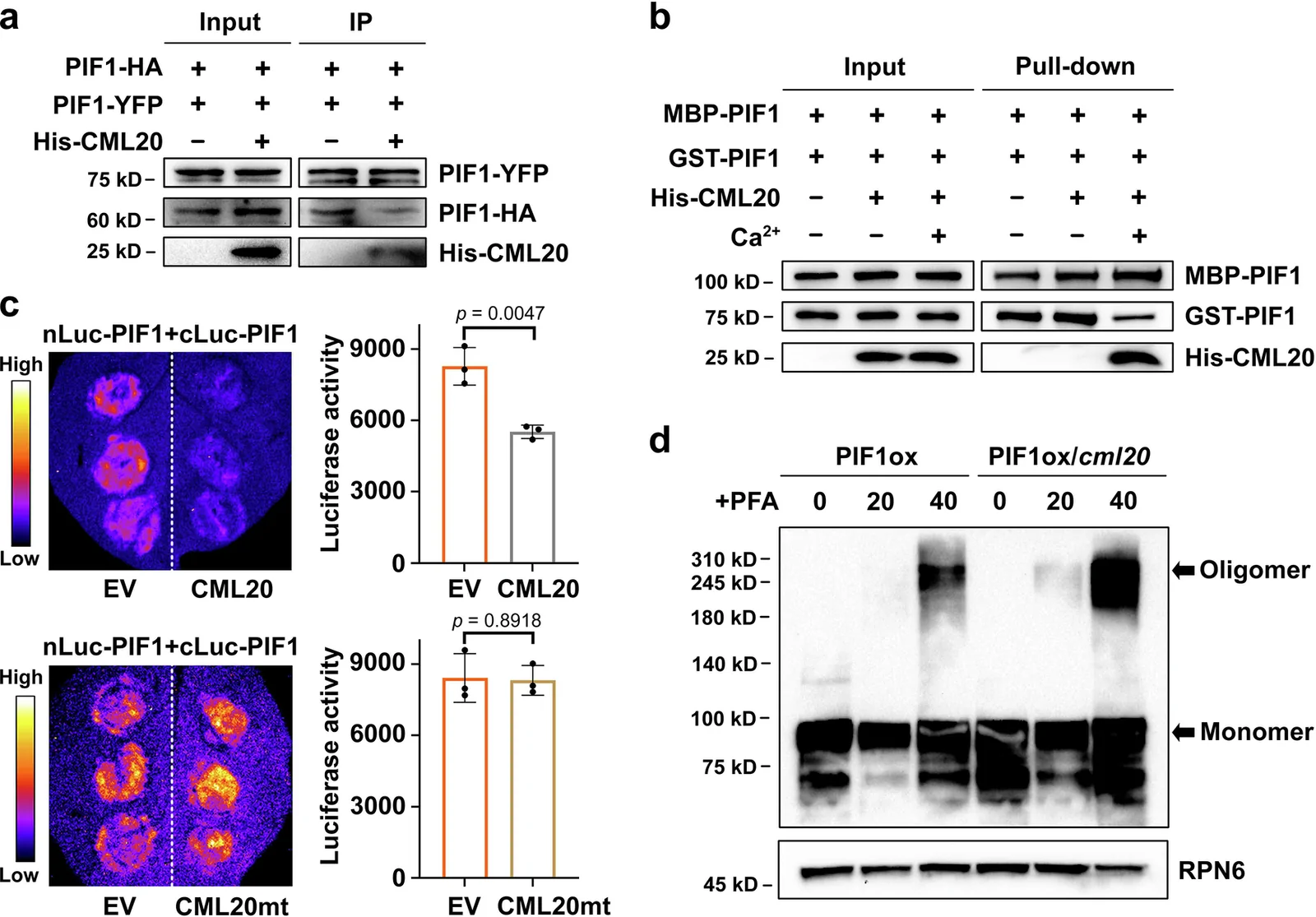
CML20 inhibits PIF1 self-interaction and oligomerization. **a** Co-IP in HEK293T cells showing that CML20 reduces PIF1 self-interaction. PIF1-YFP and PIF1-HA were expressed in HEK293T cells, and purified His-CML20 was added to cell extracts for incubation. Immunoprecipitation was performed using anti-GFP nanobody agarose beads. **b** In vitro pull-down showing Ca^2+^-dependent inhibition of PIF1 self-association by CML20. Equal amounts of purified recombinant MBP-PIF1 and GST-PIF1 proteins were incubated with or without His-CML20 in the presence (+) or absence (−) of Ca^2+^. Amylose resin was used to pull-down MBP-PIF1 and associated proteins. **c** LCI assays in tobacco leaves showing that CML20, but not CML20mt, reduces PIF1 self-interaction. Full-length PIF1 was fused to nLuc or cLuc, and *35S*:*CML20* (CML20, top) or *35S*:*CML20mt* (CML20mt, bottom) was co-expressed as indicated. Empty vectors (EV) were negative controls. Data are means ± SD; *n* = 3 biological replicates. The *p*-value was determined by using a two‑sided Student’s *t*-test. **d** In vivo chemical crosslinking showing that PIF1 forms oligomers and that *CML20* mutation enhances PIF1 oligomerization. Three-day-old etiolated seedlings of PIF1ox and PIF1ox/*cml20* were crosslinked with PFA for the indicated times. Blots were probed with anti-Myc and RPN6 was used as loading control. Source data and the exact *p*-values are provided in the Source Data file.

bHLH proteins commonly dimerize and form higher-order oligomers via their bHLH domains to facilitate stable DNA binding^62,63^. Since CML20 binds PIF1 through the C-terminus containing bHLH region, we were wondering whether CML20 modulates PIF1 self-association. In HEK293T cells, co-immunoprecipitation of PIF1-YFP recovered PIF1-HA, indicating PIF1-PIF1 self-interaction (Fig. 5a). Co-expression of CML20 markedly reduced the amount of co-precipitated PIF1-HA without altering input levels (Fig. 5a). These results suggest that CML20 suppresses PIF1 self-interaction. Consistently, in pull-down assays, recombinant MBP-PIF1 proteins bound GST-PIF1, and addition of Ca^2+^-bound CML20 dramatically impaired the self-interaction of PIF1, whereas Ca^2+^-free CML20 exerted no obvious effect (Fig. 5b). These results suggest that CML20 inhibits PIF1 self-interaction in a Ca^2+^-dependent manner.

We next tested the effects of CML20 on PIF1 self-interaction *in planta* using LCI assays. Co-expression of nLuc-PIF1 and cLuc-PIF1 produced strong luminescence in tobacco leaves, whereas co-expression of CML20 significantly reduced the complemented signal (Fig. 5c). In contrast, the Ca^2+^-binding-deficient CML20mt did not affect the complemented signal (Fig. 5c), indicating that inhibition of PIF1 self-interaction in vivo requires CML20’s Ca^2+^-binding capacity.

To further assess CML20’s effects on higher-order assembly of PIF1, we genetically crossed a PIF1-Myc overexpression line (PIF1ox) into *cml20* and performed paraformaldehyde (PFA) chemical crosslinking. Immunoblot analysis revealed comparable levels of PIF1-Myc protein in imbibed seeds of PIF1ox and PIF1ox*/cml20* under both control and stress conditions, indicating that CML20 does not regulate PIF1 stability (Suppl. Fig. 8). After PFA crosslinking, PIF1-Myc formed higher-molecular-weight species migrating at approximately 310 kDa, consistent with higher-order oligomers (Fig. 5d). Notably, these oligomeric bands were increased in the *cml20* background (Fig. 5d), indicating that loss of *CML20* enhances PIF1 oligomerization. Together, these results demonstrate that CML20 inhibits PIF1 self-interaction and higher-order oligomerization.

### CML20 impairs PIF1’s DNA binding capacity and transcriptional activity

**Fig. 6:**
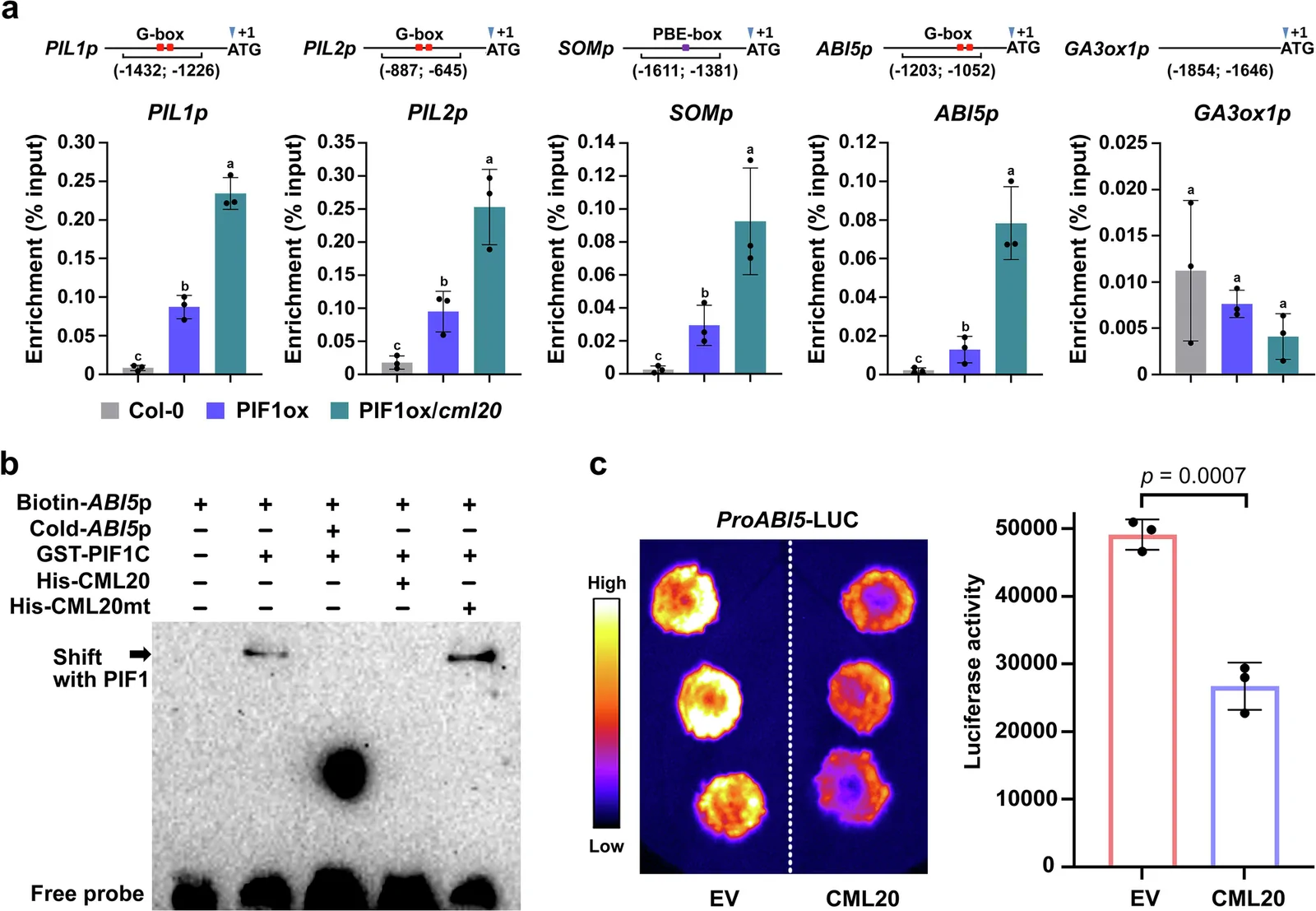
CML20 impairs PIF1 DNA binding and transcriptional activity. **a** ChIP-qPCR showing PIF1 occupancy at target gene promoters in imbibed seeds of Col-0, PIF1ox and PIF1ox/*cml20*. Data are means ± SD; *n* = 3 technical replicates. One-way ANOVA followed by Tukey’s multiple comparisons test; different letters indicate significant differences (*p* < 0.05). **b** EMSA showing that CML20 inhibits PIF1 binding to the *ABI5* promoter in a Ca^2+^-dependent manner. A biotin-labeled *ABI5* promoter probe (−1177 to −1136 upstream of ATG) containing G-box was used; an unlabeled probe served as a cold competitor. Arrow indicates the PIF1-DNA complex. **c** Images (left) and quantitative analysis (right) of luciferase bioluminescence showing CML20 represses PIF1 transcriptional activity *in planta*. A luciferase reporter driven by the *ABI5* promoter was co-expressed with full-length PIF1 in the presence (CML20) or absence (empty vector, EV) of CML20. Data are means ± SD; *n* = 3 biological replicates. The *p*-value was determined by using a two‑sided Student’s *t*-test. Source data and the exact *p*-values are provided in the Source Data file.

Given that CML20 inhibits PIF1 self-association and oligomerization, we asked whether it affects PIF1-DNA binding. To this end, we performed chromatin immunoprecipitation followed by quantitative real-time PCR (ChIP-qPCR) in imbibed seeds from PIF1ox and PIF1ox/*cml20*. The results showed that PIF1 was enriched at the promoters of known targets, including *PHYTOCHROME-INTERACTING FACTOR-LIKE 1* (*PIL1*), *PIL2*, *SOMNUS* (*SOM*), and *ABSCISIC ACID INSENSITIVE 5* (*ABI5*) (Fig. 6a). Notably, PIF1 occupancy at these loci was significantly higher in the *cml20* background (Fig. 6a), indicating that CML20 negatively regulates PIF1 binding to target promoters in vivo.

We next assessed direct effects of CML20 on PIF1-DNA binding using electrophoretic mobility shift assay (EMSA) with an *ABI5* promoter fragment. Recombinant PIF1 bound the probe, and the binding was abolished by excess unlabeled competitor, confirming the specificity of the protein-DNA complex (Fig. 6b). Addition of CML20 markedly reduced the PIF1-DNA binding, whereas the Ca^2+^-binding-defect CML20mt failed to inhibit it (Fig. 6b). These data indicate that CML20 sequesters PIF1 from binding DNA in a Ca^2+^-dependent manner.

Finally, we examined transcriptional output in a transient reporter assay in tobacco leaves. The luciferase reporter gene driven by the promoters of *ABI5* were activated by PIF1, and co-expression of CML20 significantly decreased reporter activity (Fig. 6c). Taken together, these results demonstrate that CML20 attenuates PIF1 occupancy at target promoters and represses its transcriptional activity.

### PhyB interacts with CML20 independent of Ca^2+^ or photoactivation

**Fig. 7:**
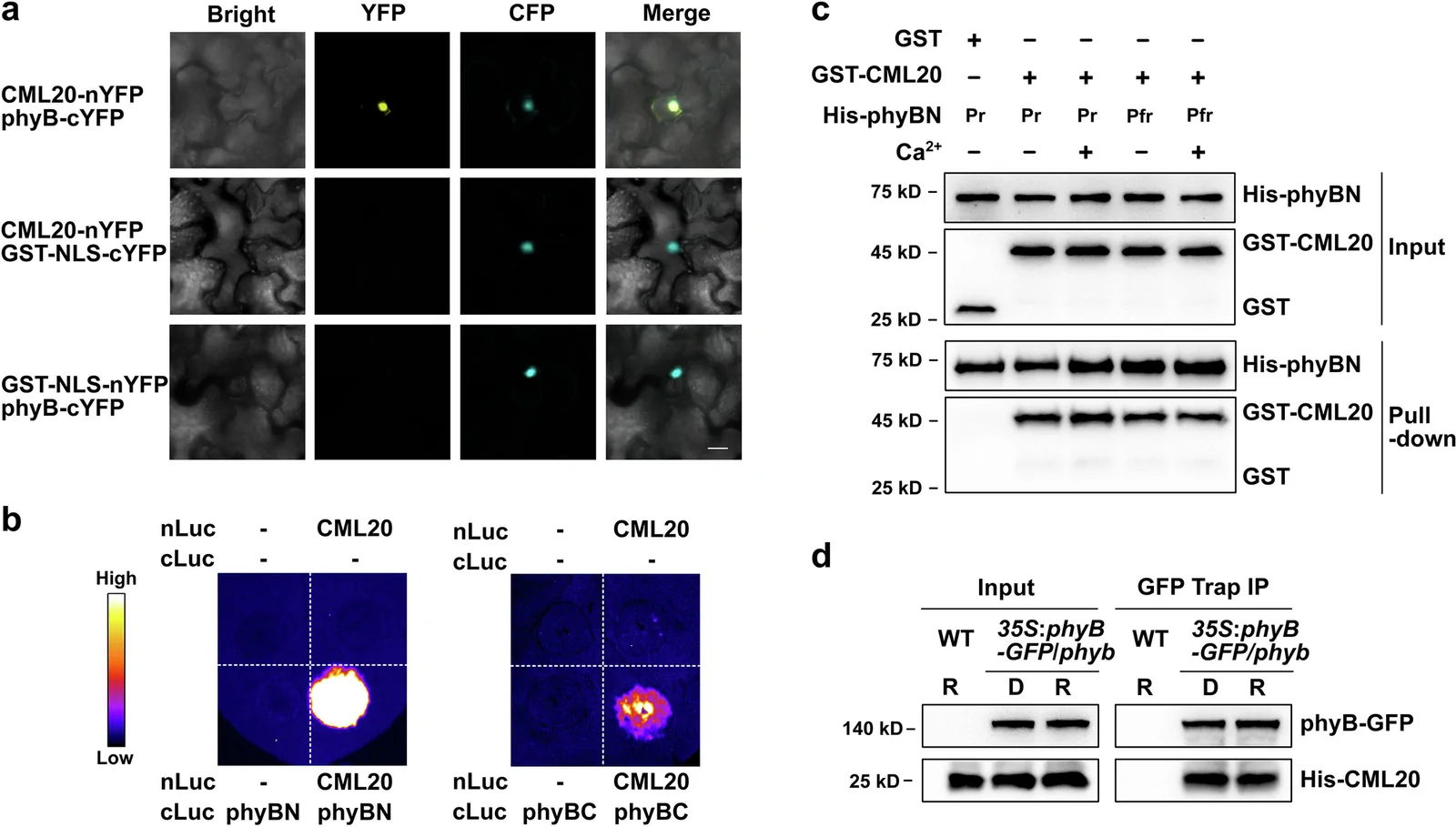
PhyB interacts with CML20 independent of Ca^2+^ and photoactivation. **a** BiFC assays showing interaction between CML20 and phyB in the nucleus of tobacco epidermal cells. GST-NLS serves as negative control. H2A-CFP marks nuclei. Scale bar = 20 μm. **b** LCI assays showing the interactions between phyB and CML20 in tobacco leaves. Full-length CML20 was fused to nLuc. The N-terminal (phyBN) or C-terminal (phyBC) fragments of phyB were fused to cLuc. Empty vectors are negative controls. **c** In vitro pull-down showing CML20 interacts with phyB Pr and Pfr conformers irrespective of Ca^2+^. His-phyBN (Pr or Pfr) were incubated with GST-CML20 or GST alone (negative control) in the presence (+) or absence (–) of Ca^2+^. Ni-NTA agarose was used to pull-down His-phyBN and associated proteins. **d** Semi-in vivo Co-IP showing that phyB-CML20 interactions occur regardless of light. *35S:phyB-GFP*/*phyb* and WT (Ler) seeds were imbibed in darkness for 24 h, then either irradiated with red light or maintained in darkness for 30 min. Equal amounts of purified His-CML20 proteins were incubated with seed protein extraction. Immunoprecipitation was performed using anti-GFP nanobody agarose beads. Source data are provided in the Source Data file.

As PIF1 is a phyB-interacting protein, we investigated whether CML20 associates with phyB. We first examined the interaction by BiFC in tobacco leaves. Co-expression of CML20-nYFP with phyB-cYFP produced nuclear YFP fluorescence, while no signal was detected in negative controls pairing with nuclear-localized GST-NLS (Fig. 7a), indicating specific nuclear-localized interaction between CML20 and phyB.

In addition, we performed LCI assays with nLuc-CML20 co-expressed with cLuc fusions of phyB’s N-terminal photosensory module or C-terminal output module (cLuc-phyBN or cLuc-phyBC). Reconstituted luminescence was detected in combinations with both phyBN and phyBC (Fig. 7b), showing that CML20 binds both the N- and C-terminal modules of phyB.

To further determine the conformer dependence for phyB-CML20 interactions, we conducted the pull-down assays. The results showed that GST-CML20 bound His-phyBN recombinant proteins in both its photo-inactive (Pr) and photoactive (Pfr) states (Fig. 7c). In addition, binding occurred with both Ca^2+^-free and Ca^2+^-binding GST-CML20, whereas GST alone did not pull down His-phyBN as negative control (Fig. 7c). These results suggest that the interaction between phyB and CML20 is independent of photo-activation or Ca^2+^.

Semi-in vivo co-immunoprecipitation from Arabidopsis further confirmed the light independence of CML20-phyB interaction. Purified His-CML20 proteins added to extracts from *35S*:*phyB–GFP*/*phyb-5* (PBG) were co-immunoprecipitated with phyB-GFP using GFP-Trap from both darkness and red light-treated samples, whereas no His-CML20 was recovered from WT (Ler) extracts lacking phyB-GFP (Fig. 7d). Together, these data show that phyB interacts with CML20 in the nucleus independently of Ca^2+^ or photoactivation.

### PhyB and Ca^2+^ independently facilitates the PIF1-CML20 interaction

**Fig. 8:**
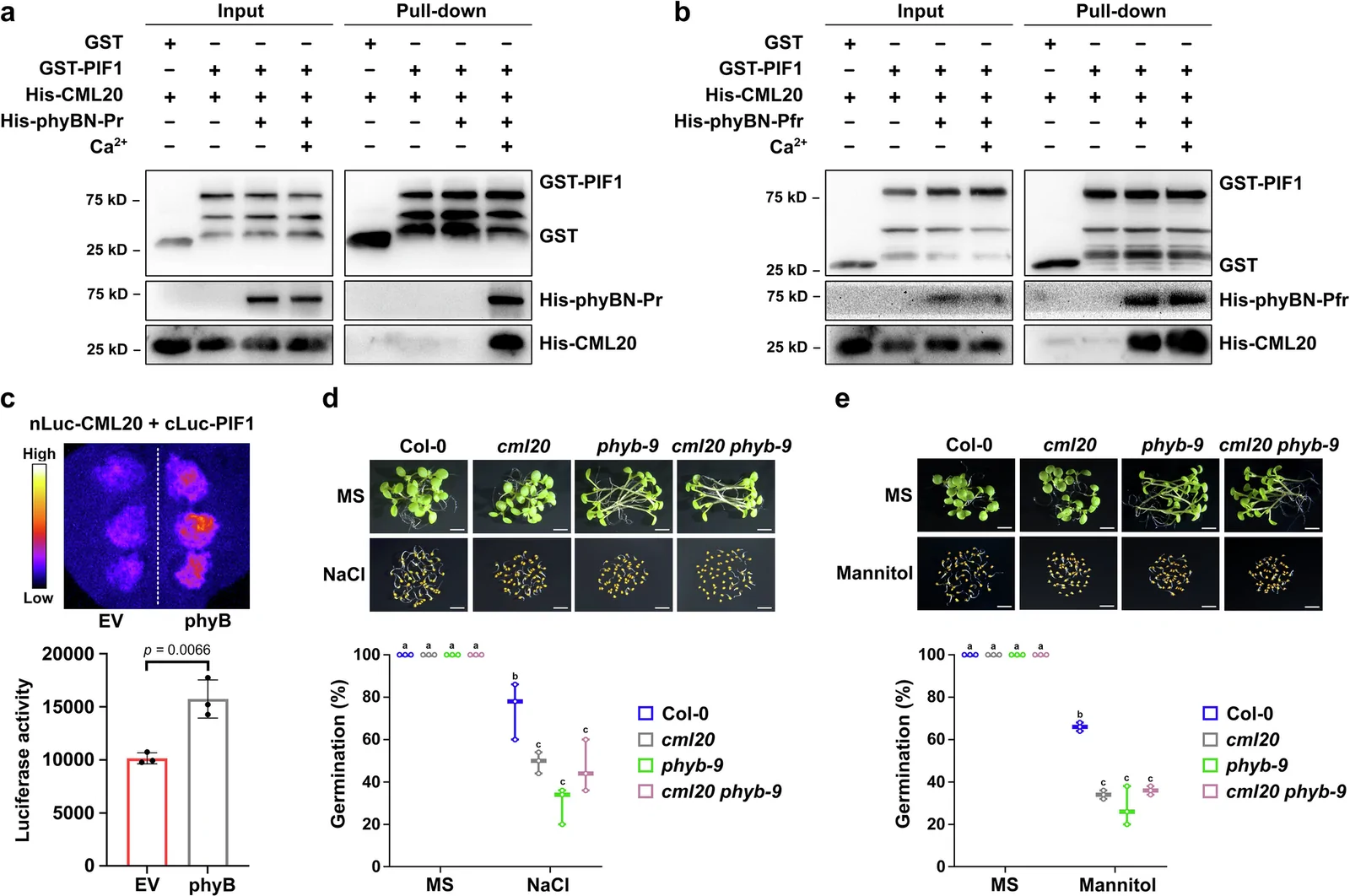
PhyB facilitates PIF1-CML20 interaction. **a**, **b** In vitro pull-down showing the effects of phyB-Pr (**a**) or phyB-Pfr (**b**) on PIF1-CML20 interaction. Purified His-phyBN was incubated with PCB in darkness for 30 min, and irradiated with far-red or red light to generate the Pr or Pfr form, respectively. His-phyBN-Pr (**a**) or His-phyBN-Pfr (**b**) was then incubated with a mixture of His-CML20 and GST-PIF1 in the presence (+) or absence (–) of Ca^2+^. Anti-GST nanobody agarose beads were used to pull down GST-PIF1 and associated His-phyBN and His-CML20. **c** LCI assays in tobacco leaves showing that phyB promotes CML20-PIF1 interaction in vivo. Full-length CML20 and PIF1 were fused to nLuc or cLuc, respectively. phyB was co-expressed under the *35S* promoter. Empty vectors (EV) are negative controls. Data are means ± SD; *n* = 3 biological replicates. The *p*-value was determined by using a two‑sided Student’s *t*-test. **d**, **e** Representative images (top) and germination rates (bottom) of Col-0, *cml20*, *phyb-9* and *cml20 phyb-9* under salinity (**d**) or osmotic (**e**) stress. Seeds were sown on MS medium containing 125 mM NaCl (**d**) or 300 mM mannitol (**e**), were stratified at 4 °C for 3 d in darkness and incubated in continuous white light for 5 d. Scale bar, 2 mm. *n* = 3 biological replicates. Two-way ANOVA followed by Tukey’s multiple comparisons test; different letters indicate significant differences (*p* < 0.05). Source data and the exact *p* values are provided in the Source Data file.

Since PIF1 interacts with the photoactive phyB, and CML20 also binds to phyB, we tested whether phyB, PIF1, and CML20 form a protein complex. In vitro pull-down assays revealed that neither inactive His-phyBN-Pr nor His-CML20 in the absence of Ca^2+^ were efficiently pulled down by GST-PIF1 (Fig. 8a), consistent with the light-dependent phyB-PIF1 interaction and Ca^2+^-dependent CML20-PIF1 binding. Upon the addition of Ca^2+^, strong interactions between CML20 and PIF1 were induced (Fig. 8a). Notably, His-phyBN-Pr proteins were also pulled down by GST-PIF1 (Fig. 8a), suggesting that Ca^2+^-activated CML20 bridges the formation of the phyB-CML20-PIF1 protein complex.

When phyB was photoactivated to the Pfr form (His‑phyBN‑Pfr), GST‑PIF1 bound to it independently of Ca^2+^ (Fig. 8b). Interestingly, the presence of His-phyBN-Pfr enhanced the association between GST-PIF1 and His-CML20 even in the absence of Ca^2+^, and this interaction was further strengthened by the addition of Ca^2+^ (Fig. 8b). These results indicate that phyB, CML20, and PIF1 each interact individually, and that their complex formation is cooperatively enhanced by light activation of phyB and Ca^2+^-supplemented conditions.

Next, we examined the effect of phyB on PIF1-CML20 interaction *in planta* by expressing nLuc-CML20 and cLuc-PIF1 in tobacco leaves. Co-expression of phyB significantly increased the complemented luciferase signals resulting from CML20-PIF1 interaction (Fig. 8c). Collectively, these results demonstrate that phyB promotes the CML20-PIF1 interaction in vitro and in vivo.

### PhyB and CML20 act in the same genetic pathway to promote stress-adapted germination

To genetically define the relationship between *phyB* and *CML20* in stress-adapted germination, we compared the germination of WT, *cml20*, *phyb-9*, and *cml20 phyb-9* seeds across a range of NaCl (0–175 mM) and mannitol (0–400 mM) concentrations. Under all tested stress levels, both *cml20* and *phyb-9* single mutants exhibited similarly reduced germination compared with WT (Fig. 8d, e, Suppl. Fig. 9). Notably, the *cml20 phyb-9* double mutant showed no additional germination defect compared to that of either single mutant (Fig. 8d, e, Suppl. Fig. 9). These genetic data indicate that phyB and CML20 function within the same pathway to promote seed germination under salt and osmotic stress, supporting a model in which phyB facilitates CML20-mediated repression of PIF1 activity.

Finally, to rule out potential confounding effects from medium composition, we compared germination responses on three different media: full-strength MS with 1% sucrose, half-strength MS with 1% sucrose, and half-strength MS without sucrose. The same relative phenotypic trends in response to NaCl and mannitol stress were observed across all media conditions (Suppl. Figs. 10, 11). These results confirm that the antagonistic roles of PIF1 and CML20, as well as the epistatic relationships between CML20 and PIF1 or phyB, are not attributable to differences in osmotic potential or nutrient availability.

## Discussion

**Fig. 9:**
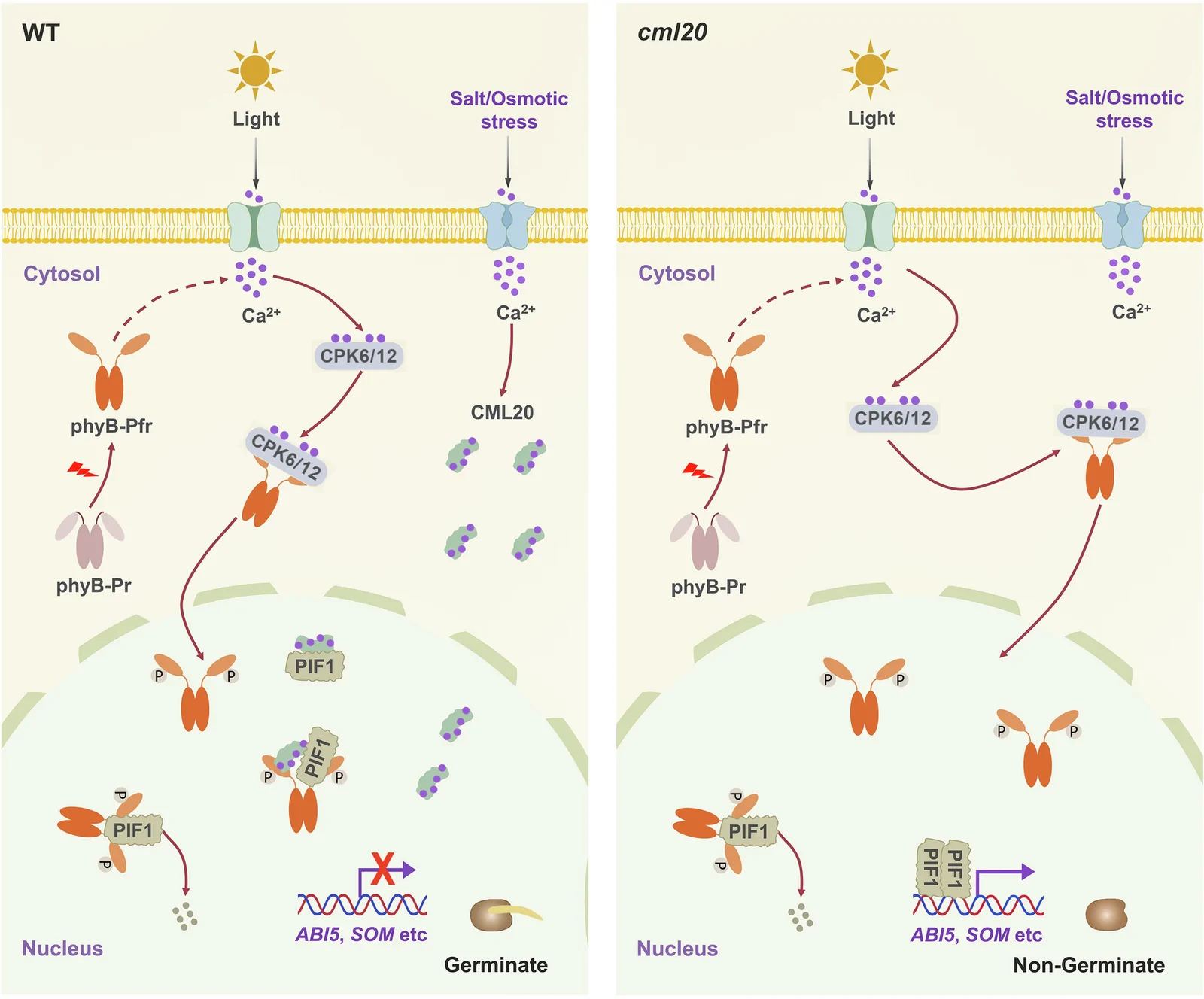
Working model of the phyB-CML20-PIF1 module in stress-adapted germination. Photo-activated phyB (Pfr form) translocates into the nucleus in a CPK-dependent manner, where it interacts with PIF1 and promotes PIF1 degradation. In WT seeds, salt or osmotic stress-induced Ca^2+^ increases activate the Ca^2+^ sensor CML20. Activated CML20 binds to PIF1, inhibiting PIF1 oligomerization and DNA-binding capacity, thereby relieving PIF1-mediated repression of germination. PhyB‑Pfr further facilitates the assembly of the CML20‑PIF1 complex, providing a rapid and reversible mechanism to fine‑tune residual PIF1 activity under stress. In the *cml20* mutant, loss of CML20-mediated repression leads to sustained PIF1 transcriptional activity, resulting in reduced germination under stress conditions.

Seed germination is a critical developmental transition that is influenced by various environmental factors, with light serving as a key promoter of this process^6^. Abiotic stresses, however, generally inhibit germination, a process that is primarily controlled by the balance between abscisic acid (ABA) and gibberellin (GA)^64,65^. From an ecological perspective, it is vital for seeds to optimize germination under stress conditions to avoid missing the opportunity to enter the new life cycle. From an agronomic standpoint, salinity and osmotic stresses are major obstacles to plant establishment, making the enhancement of germination under these stress conditions a critical goal^45^. In this study, we uncover a Ca^2+^-sensor-based mechanism that coordinates light and salt/osmotic stress to regulate seed germination (Fig. 9). Upon stress-induced increases in Ca^2+^, the calmodulin-like protein CML20 directly interacts with PIF1, inhibiting its self-association, oligomerization, and DNA-binding activity, thereby alleviating its repression on germination. Additionally, phyB interacts with both CML20 and PIF1, promoting the assembly of the CML20-PIF1 complex and further reducing PIF1 activity. By facilitating this interaction, phyB ensures that Ca^2+^ signals are effectively translated to relieve PIF1-mediated repression. In *cml20* mutants, where the repression of seed germination by PIF1 cannot be efficiently released, low germination frequencies under stress conditions occur. Our findings suggest that the CML20-PIF1 module optimizes seed germination under challenging conditions. This finely tuned control offers potential strategies for improving seed establishment in saline or drought soil environments.

PIF1 is a master transcription factor that regulates seed germination, with its activity tightly controlled by light signals^6,22,28^. In darkness, PIF1 proteins are stabilized and accumulate abundantly^25,30,35^. Conventionally, phyB directly interacts with PIFs upon light exposure, triggering their phosphorylation and degradation^25,32,34,36,37^. Moreover, phyB has been shown to inhibit PIF’s transcriptional activity^66,67^, highlighting the importance of non-proteolytic regulation. The phyB-mediated rapid protein degradation of PIF1 during the dark-to-light transition therefore serves as an essential “on switch” to relieve its repression and initiate germination. Once seeds are exposed to continuous light, PIF1 levels are substantially reduced but maintain a low, steady-state. These residual PIF1 proteins retain repressive activity, which becomes particularly relevant when seeds encounter abiotic stress. We found that under salt or osmotic stress conditions, phyB associates with both CML20 and PIF1 and facilitates their interaction, thereby inhibiting PIF1’s DNA-binding and transcriptional activity. Thus, the phyB-CML20-PIF1 module provides a reversible mechanism to modulate the activity of the remaining PIF1 pool, allowing seeds to maintain germination competence under stress conditions.

Several light-responsive regulators, such as HFR1, LEUNIG (LUG) and its homolog LUH, REVEILLE1/2 (RVE1/2), and HECATE1/2 (HEC1/2), have been implicated in fine-tuning PIF1 activity^38–41^. Recently, the B-box protein BBX32 has been identified as a negative regulator of PIF1 in response to imbibition signals^43^. In our study, we reveal that CML20, a Ca^2+^-binding protein, serves as a salt- and mannitol-induced regulator of PIF1. Salt or osmotic stress triggers a Ca^2+^ increase in imbibed seeds, and this Ca^2+^ elevation enhances the CML20-PIF1 interaction, supporting the physiological relevance of this regulatory module. In addition, phyB may serve as a scaffold that brings CML20 and PIF1 into closer proximity, thereby increasing their encounter frequency; alternatively, phyB may exert allosteric effects on PIF1 or CML20, altering their conformations and enhancing mutual recognition. Together, these findings emphasize that controlling transcriptional activity provides a flexible mechanism to integrate multiple signals and dynamically regulate germination. While proteolytic mechanisms may confer robustness to the light-triggered transition, the non-proteolytic mechanisms offer reversibility, enabling plants to rapidly adjust in fluctuating environments.

Typically, Ca^2+^ signals are converted into phosphorylation responses through CaM-dependent kinases in animals (e.g., CaMKII/CaMKIV) and through plant-specific Ca^2+^-regulated kinases and phosphatases (e.g., CPKs and CIPKs), but growing evidence extends Ca^2+^ decoding to direct transcriptional control^49,68–70^. In this context, CML20 acts as a Ca^2+^ sensor that binds the bHLH domain of PIF1 in a Ca^2+^-dependent manner, thereby restraining PIF1’s DNA-binding capacity. Notably, previous studies have shown that mammalian bHLH transcription factors exhibit reduced DNA binding upon direct CaM association^71^. In plants, a specific CaM isoform has been shown to enhance the DNA-binding activity of AtMYB2^72^, illustrating that Ca^2+^ sensors can either inhibit or potentiate bHLH transcription factor-DNA interactions, depending on the context. Given the prevalence of EF-hand sensors and bHLH factors in plants, analogous Ca^2+^-CML control points may exist for other bHLH transcription factors that govern development and stress responses. Future high-resolution structural studies will be necessary to define how Ca^2+^-bound CML20 interacts with the PIF1 bHLH domain and impedes dimerization and DNA binding^73^. Additionally, specificity is conferred by distinct CaM/CML isoforms through their Ca^2+^-binding affinities, subcellular localization, partner preferences, and expression patterns^55,74,75^. Consistent with this, CML20 shows high identity to its closest members but displays a divergent expression pattern, highlighting the role of expression dynamics in specifying Ca^2+^ sensor function.

Although plants respond to individual environmental stimuli with considerable specificity, optimal growth and survival rely on integrating multiple cues. Substantial evidence shows that light signaling coordinates with abiotic stress pathways across organs and developmental stages^58,59,76–82^. As a versatile second messenger, Ca^2+^ is increasingly recognized as integral to light signaling. Recent work shows that red light elicits rapid cytosolic Ca^2+^ transients via phyB, which are decoded by Ca^2+^-dependent protein kinases CPK6 and CPK12. Upon Ca^2+^ activation, CPK6/12 directly interact with and phosphorylate photoactivated phyB to initiate its nuclear import^17^. Also, the Ca^2+^-responsive transcription factor CAMTA3 helps maintain phyB abundance^83^, and CAM7 cooperates with HY5 to bind the *HY5* promoter in promoting photomorphogenesis^84,85^. In this study, we add a seed-focused Ca^2+^ sensor to this network by showing that CML20 links light and stress signaling pathways during germination. Under salt and osmotic stress imbibed seeds face reduced water potential and disrupted ion homeostasis that generally inhibit germination^65,86^. In our model, stress-evoked Ca^2+^ transients activate CML20, which binds PIF1 to limit its dimerization and DNA binding, while phyB promotes assembly of the CML20-PIF1 complex. This dual control enables seeds to capitalize on favorable light conditions while buffering the inhibitory effects of transient stresses, thus preventing germination failure in illuminated yet challenging environments. Together, these findings emphasize that light and Ca^2+^ signaling are tightly integrated at multiple levels, from photoreceptors to transcription factors, enabling precise perception of and adaptation to environmental cues.

## Methods

### Plant material and growth conditions

The Columbia (Col-0) ecotype of Arabidopsis thaliana was used as the wild type, unless otherwise specified. The *phyb-9*^16^, *pif1*^87^, *pif3*^87^, *pif4*^87^, *pif5*^87^, *35S:phyB-GFP*/*phyb-5* (PBG)^88^, PIF1ox^22^ and AEQ^61^ were previously reported. The *phyb-9* mutant was backcrossed into the Col-0 background, and genotyping confirmed the presence of the wild-type *VENOSA4* gene^89^. The Arabidopsis T-DNA insertion mutants *cml20* (SALK_079974) and *cml20-2* (CS925331) were obtained from Arabidopsis Biological Resource Center (ABRC)^60,90^. The *cml20 pif1*, *cml20 phyb-9*, and PIF1ox/*cml20* were generated by genetic crossing.

For generating *35S*:*CML20-Myc*/*cml20* or *35S*:*CML20-YFP*/*cml20* transgenic Arabidopsis plants, the coding sequences of full-length CML20 were amplified from Arabidopsis cDNA and cloned into pENTR4 vector (Invitrogen, A10465) as entry construct, and transferred by LR recombination into pGWB-617 or pGWB-641, respectively^91^. For generating *35S*:*PIF1-Myc*/*pif1* transgenic Arabidopsis plants, the coding sequences of full-length PIF1 were amplified from Arabidopsis cDNA and cloned into pENTR4 vector (Invitrogen) as entry construct, and transferred by LR recombination into pGWB-17. *Agrobacterium tumefaciens* (GV3101) carried the indicated construct was transformed into Arabidopsis plants using floral-dip method.

For *Arabidopsis thaliana*, surface-sterilized seeds were plated on Murashige and Skoog (MS) medium (4.3 g/L MS salts, 10 g/L sucrose, 0.28 g/L MES, and 8 g/L agar, pH = 5.8), and then were grown in growth chamber at 22 °C under a 16 h light/8 h dark cycle with 40–60% relative humidity. For *Nicotiana benthamiana*, seeds were sown on soil, and grown in growth chamber at 25 °C under a 16 h light/8 h dark cycle.

### Bacterial, cell and fungal strains

*Escherichia coli* stains (DH5ɑ and BL21) and *Agrobacterium tumefaciens* stains (GV3101 and GV2260) were cultured in LB medium with appropriate antibiotics at 37 °C and 28 °C, respectively. Human embryo kidney cells (HEK293T) (Procell, CL-0005) were maintained at 37 °C with 5% CO₂ in DMEM high glucose (Gibco) supplemented with 10% fetal bovine serum (Gibco) and 1% penicillin-streptomycin (HyClone). The yeast EGY48 strain were incubated at 30 °C in SD-deficient selective medium.

### Seed germination assay

Germination assays were adapted from previously described protocols with the modifications detailed as follows^59,92,93^. For each experimental batch, all seeds were grown and harvested side by side. Seeds were harvested and stored at room temperature for two months before use. Seeds were surface-sterilized and sown on MS medium (or half-strength MS or half-strength MS without sucrose, as specified in the figure legends) supplemented with NaCl or mannitol as indicated. The seeds were stratified at 4 °C in darkness for 3 days, and exposure to white light for 8 h. Plates were incubated at 22 °C under continuous white light (60 μmol m^−2^ s^−1^) or darkness for 5 days. Germination rates were scored at indicated time points. Each experiment used 50 seeds per genotype, with three biological replicates for statistical analysis.

### PhyB-dependent seed germination assay

Seeds were harvested and stored at room temperature for two months prior to use. After surface sterilization, seeds were sown on MS medium containing the indicated concentrations of NaCl or mannitol. To inactivate phyB, seeds were exposed to far-red light (20 μmol m^−2^s^−1^) for 5 min. For the phyB-OFF condition, seeds were subsequently incubated in darkness at 22 °C for 5 d. For the phyB-ON condition, following the same far-red treatment, seeds were irradiated with red light (40 μmolm^−2^s^−^^1^) for 5 min to activate phyB, and then transferred to darkness for 5 days at 22 °C. Germination rates were scored after the 5-day incubation. Each experiment included 50 seeds per genotype and was performed with three biological replicates for statistical analysis.

### RNA extraction, and RT-qPCR

Tissues were ground in liquid nitrogen, and total RNA was extracted using the Spectrum Plant Total RNA Kit (Sigma-Aldrich, STRN50-1KT) with on-column DNase I digestion (Sigma-Aldrich, DNASE70-1SET). RNA quality and concentration were assessed by agarose gel electrophoresis and spectrophotometry. cDNA was synthesized from 1 μg RNA using ReverTra Ace qPCR RT Master Mix (Toyobo, FSQ-301). RT-qPCR was performed using Hieff qPCR SYBR Master Mix (Yeason, 11202ES08) on ABI Q6 Pro with three technical replicates. Gene-specific primers were listed in Suppl. Table 1.

### Yeast two-hybrid assay

The full-length *CML20* coding sequence was cloned into pLexA (Novopro, V007602) to generate BD-CML20 construct. Full-length or truncated PIF1 sequences were cloned into pB42AD (Novopro, V005332) to obtain AD-PIF1, AD-PIF1N (1–150 aa), AD-PIF1C (151–478 aa) constructs. The indicated combinations were co-transformed into yeast strain EGY48 and selected on SD/-Ura-His-Trp medium at 30 °C for 3 days. Positive interactions were identified by blue colony formation on SD/Raf/Gal-Ura-His-Trp medium containing X-Gal (5-bromo-4-chloro-3-indolyl-β-D-galactopyranoside).

### Protein expression and purification

The full-length or truncated coding region of *PIF1* were cloned into the pGEX4T-1 (GE Healthcare, 28-9545-49) or pMAL (NEB, E8201S) vector to generate GST-PIF1, GST-PIF1N (1–150 aa) or GST-PIF1C (151–478 aa), MBP-PIF1 constructs, respectively. The N-terminal region of *phyB* (1–655 aa) was cloned into pET28a to produce His-phyBN construct. The full-length *CML20* coding region was cloned into pET28a or pGEX4T-1vector to generate His-CML20 or GST-CML20, respectively. Site-directed mutagenesis (Transgen, FM201-01) was used to introduce E83A and E156A substitutions in CML20, and the mutant was cloned into pET28a (His-CML20mt). All the constructs were transformed into *E.coli* BL21 strain, and protein expression was induced with 0.5 mM IPTG for 20 h at 18 °C. Cells were harvested, lysed by sonication, and recombinant proteins were purified using Amylose Resin (NEB, E8021S), Ni-NTA agarose (QIAGEN, 30210), or Anti-GST Nanobody Magarose Beads (AlpaLifeBio, KTSM1355). His‑CML20 should be freshly prepared for the following analysis, as freeze‑thaw cycles lead to degradation.

### Immunoblot analysis

Arabidopsis samples were harvested under dim green light, frozen in liquid nitrogen, and homogenized. Proteins were extracted in buffer (50 mM Tris-HCl, pH = 6.8, 25% glycerol, 10% SDS, 2.5% 2-mercaptoethanol, 1 mM PMSF, and 0.5% bromophenol blue), vortexed, and denatured at 95 °C for 10 min. After centrifugation at 15,871 × *g* for 10 min, supernatants were separated by SDS-PAGE and transferred to PVDF membranes. Membranes were blocked with 5% milk and probed with antibodies against GFP (Abcam, ab13970, 1:2000), Myc (Sigma-Aldrich, M4439, 1:1000), ACTIN (Sigma-Aldrich, A0480, 1:50,000), and RPN6 (Abmart, PA4990, 1:8000).

### Pull-down assays

Purified recombinant proteins were incubated in binding buffer (20 mM Tris-HCl, pH = 7.5, and 150 mM NaCl) in the presence of 1 mM EGTA or 0.5 mM CaCl_2_ at 4 °C for 1 h with gentle rotation as previously reported with the modifications detailed as follows^17,94,95^. For phyB-containing assays, His-phyBN was incubated with phycocyanobilin (PCB) on ice in darkness for 30 min, and then split to exposure under far-red or red light for additional 30 min to generate the Pr or Pfr conformers, respectively. The phyB Pr or Pfr was added in binding buffer. After incubation, Ni-NTA agarose (Qiagen, 30210), Anti-GST Nanobody Magarose Beads (AlpaLifeBio, KTSM1355), or Amylose Resin (New England Biolabs, E8021S) was added for 2 h at 4 °C with rotation. Beads were washed three times with washing buffer (50 mM Tris-HCl, pH = 7.5, 10 % Glycerol, 0.1 % Triton X-100, and 1 mM EDTA). The proteins were separated by SDS-PAGE and immunoblotted with anti-MBP (NEB, E8032, 1:2000), anti-His (ABclonal, AE086, 1:2000) anti-GST (ABclonal, AE006, 1:3000) antibodies.

### Bimolecular fluorescence complementation assay (BiFC)

The indicated coding sequences were fused in frame with the N/C-terminus of yellow fluorescence protein (YFP) (PIF1-nYFP, CML20-n/cYFP, phyB-nYFP, CIP8-n/cYFP and GST-NLS-n/cYFP). H2A-CFP was used as a nuclear marker. Constructs were co-infiltrated into tobacco leaves via Agrobacterium strain GV2260. After 3 d, YFP and CFP signals were visualized by confocal microscopy (ZEISS 780 Meta).

### In vivo and semi-in vivo co-immunoprecipitation (Co-IP) assays

For co-IP in mammalian HEK293T cells, full-length *PIF1* was cloned into pcDNA3.1-YFP and pcDNA3.1-HA. PIF1-YFP and PIF1-HA were expressed in HEK293T cells and extracted with buffer (50 mM Tris-HCl, pH = 7.5, 150 mM NaCl, 1% NP40, and 1 mM PMSF), then the equal amounts of PIF1-YFP and PIF1-HA protein extracts incubated with or without purified His-CML20 for 1 h, and immunoprecipitated with anti-GFP nanobody agarose beads (AlpaLifeBio, KTSM1301).

In vivo co-immunoprecipitation (Co-IP) and semi-in vivo Co-IP assays in Arabidopsis were adapted from previously described protocols with the modifications detailed as follows^96^. For in vivo Co-IP, the imbibed *35S*:*PIF1-Myc* and *35S*:*PIF1-Myc*/*35S*:*CML20-YFP* seeds were harvested in liquid nitrogen under dim green light. Proteins were extracted in lysis buffer (50 mM Tris-HCl, pH = 7.5, 1 mM EDTA, 150 mM NaCl, 10% Glycerol, 1 mM PMSF, 0.1% Tween-20 and protease inhibitor cocktail). For semi-in vivo Co-IP, PBG and Ler seeds were imbibed for 24 h, treated with red light or kept in darkness, and extracts were incubated with purified His-CML20. Anti-GFP nanobody agarose beads (AlpaLifeBio) were added in supernatants for 2 h at 4 °C with rotation. The immunoblot analysis was performed using anti-GFP (Abcam, ab13970, 1:2000), anti-His (ABclonal, AE086, 1:2000), anti-Myc (Sigma-Aldrich, M4439, 1:3000) and anti-HA (Cell Signaling Technology, 45346, 1:1000) antibodies.

### Luciferase bioluminescence imaging (LCI)

The indicated coding sequences were cloned into pCAMBIA1300-nLuc or pCAMBIA1300-cLuc vector^97^ to generate nLuc-CML20, nLuc-CML20-mt, nLuc-PIF1, cLuc-PIF1, cLuc-PIF1N (1–150 aa), cLuc-PIF1C (151–478aa), cLuc-phyBN (1–655 aa) and cLuc-phyBC (600–1172 aa). The promoter sequence approximately 1800 bp upstream from ATG of *ABI5* were cloned into pGWB-435 vector carrying the *Luc* reporter gene. Agrobacterium strain GV2260 carrying indicated constructs was infiltrated into tobacco leaves. After 3 d, leaves were injected with 1 mM d-luciferin, and luminescence was captured using a ChampChemi 610 Plus system (SageCreation) and quantified with ImageJ.

### Aequorin-based Ca^2+^ signal imaging

For Ca^2+^ imaging, seeds of Arabidopsis transgenic lines expressing cytosolic aequorin (AEQ) were stratified at 4 °C for 24 h. After removal of the seed coat, the seeds were incubated with 40 µM coelenterazine (NanoLight Technologies, 303-10 mg) in darkness overnight. The remaining coelenterazine solution was then removed, and the seeds were treated with water, 125 mM NaCl, or 660 mM mannitol^46^. Luminescence was captured over a 3 min exposure period using a Berthold LB985 luminescence imager. The bioluminescence signal intensity was quantified using IndiGo software (Berthold Technologies).

### Electrophoretic mobility shift assay

Electrophoretic mobility shift assay was performed by Light Shift Chemiluminescent EMSA Kit (Thermo, 20148). Biotin-labeled and unlabeled probes corresponding to the *ABI5* promoter G-box were prepared. Purified GST-PIF1C (2 μg) was incubated with 20 fmol biotin-labeled probe in binding buffer (1× binding buffer, 50 ng/μL Poly dI-dC, 2.5% glycerol, 5 mM MgCl_2_ and 0.05% NP-40). For competition, 4 pmol unlabeled probe was used. The binding reactions were completed at room temperature for 20 min, and separated on 6% native polyacrylamide gels in 0.5× TBE and detected by chemiluminescence.

### Chromatin immunoprecipitation (ChIP)-qPCR

Seeds were imbibed under darkness for 1 h, given 5 min far-red light, and returned to darkness for 3 h. A total of 2 g samples were cross-linked in 1% formaldehyde under vacuum for 30 min and terminated the crosslinking reaction by glycine under vacuum for 10 min. The harvested seeds were ground into powder in liquid nitrogen, and chromatin complexes were isolated and sonicated. The sonicated chromatin complexes were precipitated with anti-Myc nanobody agarose beads (AlpaLifeBio, KTSM1306). DNA was purified and analyzed by qPCR with primers listed in Suppl. Table 1.

### Nuclear‑cytoplasmic fractionation assay

Approximately 0.5 g of imbibed seeds were harvested in liquid nitrogen. Proteins were extracted in lysis buffer (20 mM Tris-HCl, pH = 7.5, 25% Glycerol, 20 mM KCl, 2 mM EDTA, 2.5 mM MgCl_2_, 250 mM sucrose, 30 mM β-Mercaptoethanol, 1 mM PMSF, 10 μg/mL Leupeptin, 1 mM NaF, 1 mM Na_3_VO_4_, 1 mM β-glycerophosphate, 1 mM Na_2_P_2_O_4_) and mixed gently. The homogenate was filtered sequentially through nylon meshes, and the filtrate was centrifuged at 1500 × *g*. The resulting supernatant constituted the cytoplasmic fraction, and the pellet was collected as the nuclear fraction. The nuclear pellet was washed and resuspended in NRBT solution (20 mM Tris-HCl, pH = 7.5, 25% Glycerol, 2.5 mM MgCl_2_, 0.2% Triton X-100). The proteins were separated by SDS-PAGE and immunoblotted with antibodies against GFP (Abcam, ab13970, 1:2000), Myc (Sigma-Aldrich, M4439, 1:1000), ACTIN (Sigma-Aldrich, A0480, 1:50000), and Histone H3.1 (Abmart, P30266, 1:6000).

### Sequence alignment and predicted protein structure analysis

Protein sequences of CML19, CML20, CML21 and CML22 were obtained from NCBI (CML19 GI: 1063727279, CML20 GI: 1063715990, CML21 GI: 1063725093, CML22 GI: 1063708534) and aligned using DNAMAN. Structures were predicted with AlphaFold (https://www.alphafold.ebi.ac.uk) and visualized in Chimera X software.

### Quantification and statistical analysis

For two independent groups, Student’s *t*-test was conducted and the significant differences indicated as the *p*-values. For multiple comparisons, one-way or two-way ANOVA followed by Tukey’s multiple comparisons test were performed and the significant differences were denoted by different lowercase letters. *p* < 0.05. The *p* values obtained following Tukey’s multiple comparisons test are provided in the Source Data file. The sample size used for determining the mean and standard deviation was presented in figure legends.

### Accession numbers

*CML13*, At1g12310; *CML19*, At4g37010; *CML20*, At3g50360; *CML21*, At4g26470; *CML22*, At3g24110; *CML27*, AT1g18210; *CML35*, AT2g41410; *CML49*, AT3g10300; *CML50*, AT5g04170; *CAM1*, AT5g37780; *CAM3*, AT3g56800; *CAM4*, AT1g66410; *CAM5*, AT2g27030; *CAM7*, AT3g43810; *CIP8*, At5g64920; *GA3ox1*, At1g15550; *phyB*, At2g18790; *PIF1*, At2g20180; *PIL1*, At2g46970; *PIL2*, At3g62090; *SOM*, At1g03790; *ABI5*, AT2g36270; *UBQ5*, At3g62250; *ACTIN2*, At3g18780.

### Reporting summary

Further information on research design is available in the Nature Portfolio Reporting Summary linked to this article.

## Supplementary information

Supplementary Information

Reporting Summary

Transparent Peer Review file

## Source data

Source Data

## Data Availability

All data generated in this study are provided within the paper and Suppl. Information. Source data are provided with this paper.
