# Supplementary material for: CML20 integrates phytochrome and calcium signaling pathways to optimize seed germination under abiotic stress
Source: Nat Commun. 2026 Sep 4;17:10540. doi: 10.1038/s41467-026-77567-0 (PMC13642242; doi:10.1038/s41467-026-77567-0)
Supplement: Supplementary file 1 [file 41467_2026_77567_MOESM1_ESM.pdf]

Supplementary Information

CML20 integrates phytochrome and calcium signaling pathways to optimize seed germination under abiotic stress

Jing Kang[1], Jing Li[2,3], Xiaoyang Liu[1], Shuai Zhao[1], Shangwei Zhong[2,3], Hui Shi[1]*

1. College of Life Sciences, Capital Normal University, Beijing, 100048, China

2. Peking University Institute of Advanced Agricultural Sciences, Shandong Laboratory of Advanced Agriculture Sciences in Weifang, Weifang, 261325, China

3. State Key Laboratory of Gene Function and Modulation Research, Peking-Tsinghua Center for Life Sciences, School of Life Sciences, Peking University, Beijing, 100871, China

* Correspondence: H.S. (hui.shi@cnu.edu.cn)

This document contains Supplementary Figures 1 to 11, and Supplementary Table 1.

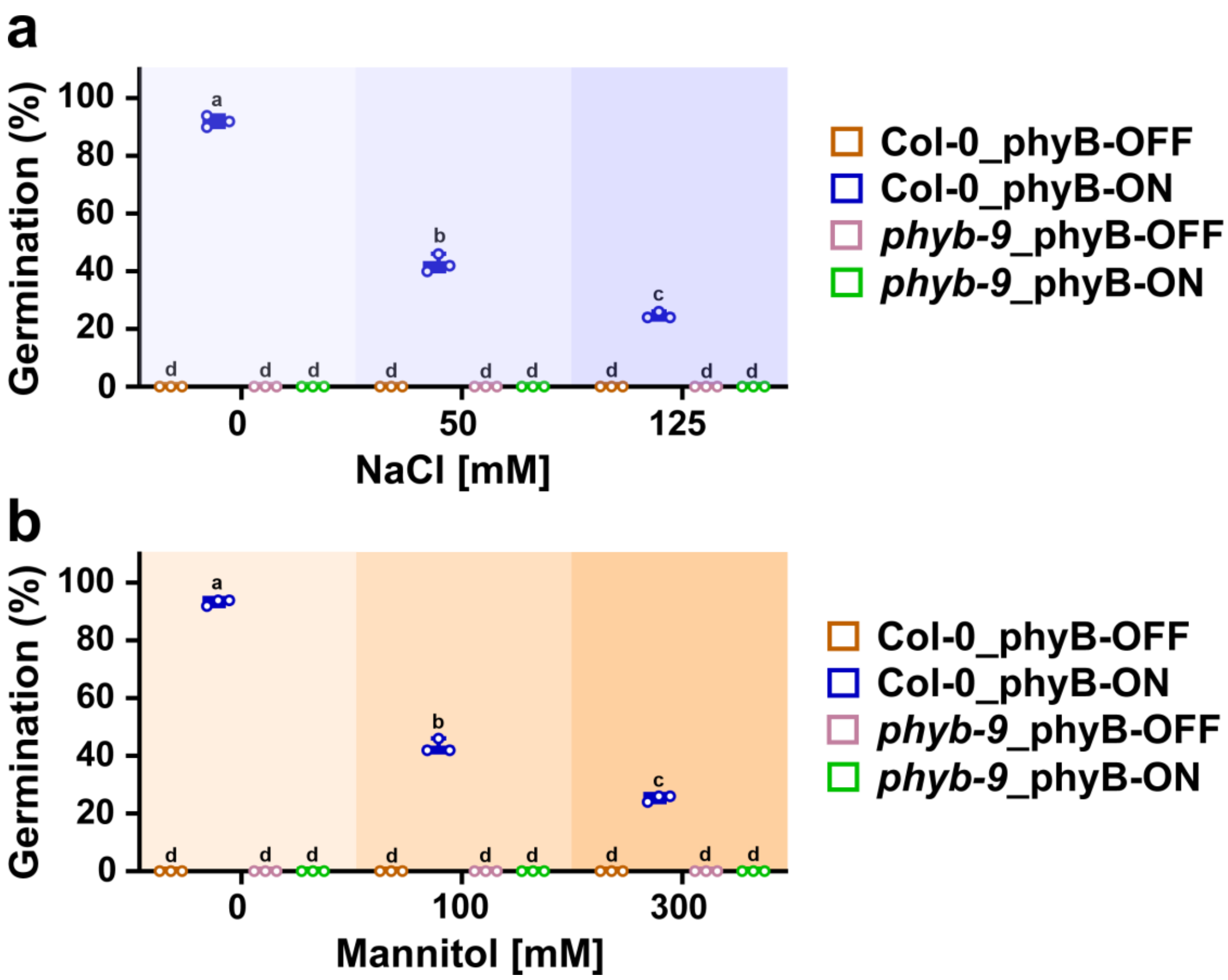

Supplementary Fig. 1. Germination of Col-0 and *phyb-9* under phyB-ON and phyB-OFF conditions in response to salt or osmotic stress.

a, b Germination rates of Col-0 and *phyb-9* seeds on MS medium supplemented with the indicated concentrations of NaCl (**a**) or mannitol (**b**) under phyB-OFF or phyB-ON conditions. Seeds were surface-sterilized and sown on medium under white light (within 1 h). For the phyB-OFF condition, seeds were irradiated with 5 min of far-red light to inactivate phyB. For the phyB-ON condition, seeds were irradiated with 5 min of far-red light followed immediately by 5 min of red light to activate phyB. After light treatments, seeds were incubated in darkness for 5 d. *n* = 3 biological replicates. Two-way ANOVA followed by Tukey's multiple comparisons test; different letters indicate significant differences ($p < 0.05$). Source data and the exact *p* values are provided in the Source Data file.

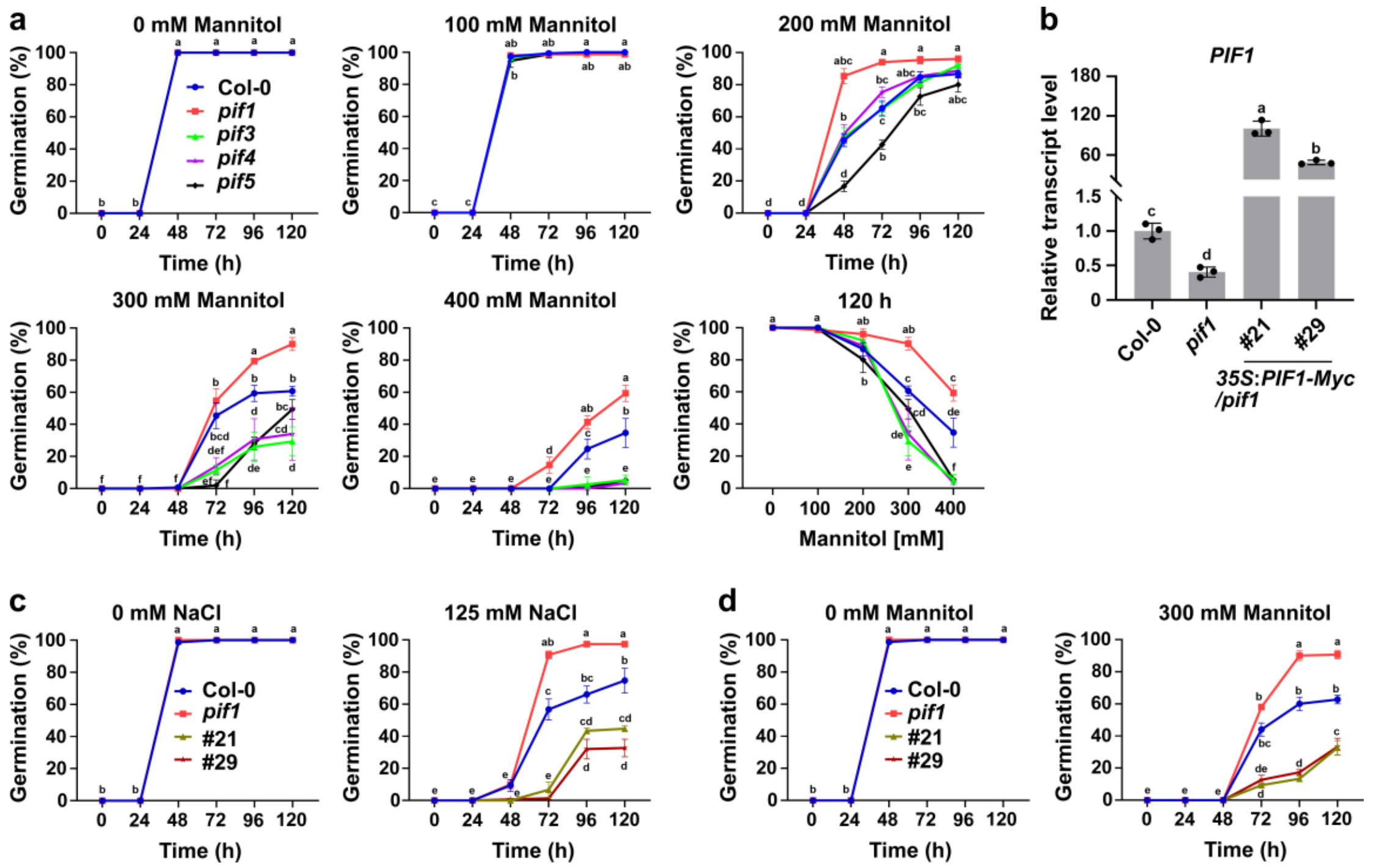

Supplementary Fig. 2. Germination of *PIF*-related mutants and transgenic lines under salt or osmotic stress.

a Time-course germination of Col-0, *pif1*, *pif3*, *pif4*, and *pif5* seeds. Seeds were sown on MS medium containing the indicated mannitol concentrations, stratified at 4 ℃ for 3 d in darkness, and incubated in continuous white light. Germination was recorded every 24 h. Data are means ± SD; n = 3 biological replicates. Two-way ANOVA followed by Tukey's multiple comparisons test; different letters indicate significant differences ($p < 0.05$).

b RT-qPCR showing *PIF1* expression in Col-0, *pif1* and two *35S*:*PIF1-Myc*/*pif1* transgenic lines. *UBQ5* was used as the internal control for normalizing transcript levels. Data are means ± SD; n = 3 technical replicates. One-way ANOVA with Tukey's test; different letters indicate significant differences ($p < 0.05$).

c, d Time-course germination of Col-0, *pif1*, and two *35S:PIF1-Myc*/*pif1* lines under salt (**c**) or osmotic (**d**) stress. Seeds were sown on MS medium containing 125 mM NaCl (**c**) or 300 mM mannitol (**d**), stratified at 4 ℃ for 3 d in darkness, and incubated in continuous white light. Germination was recorded every 24 h. #21 and #29 are independent *35S:PIF1-Myc*/*pif1* transgenic lines. Data are means ± SD; n = 3 biological replicates. Two-way ANOVA followed by Tukey's multiple comparisons test; different letters indicate significant differences ($p < 0.05$).

Source data and the exact p values are provided in the Source Data file.

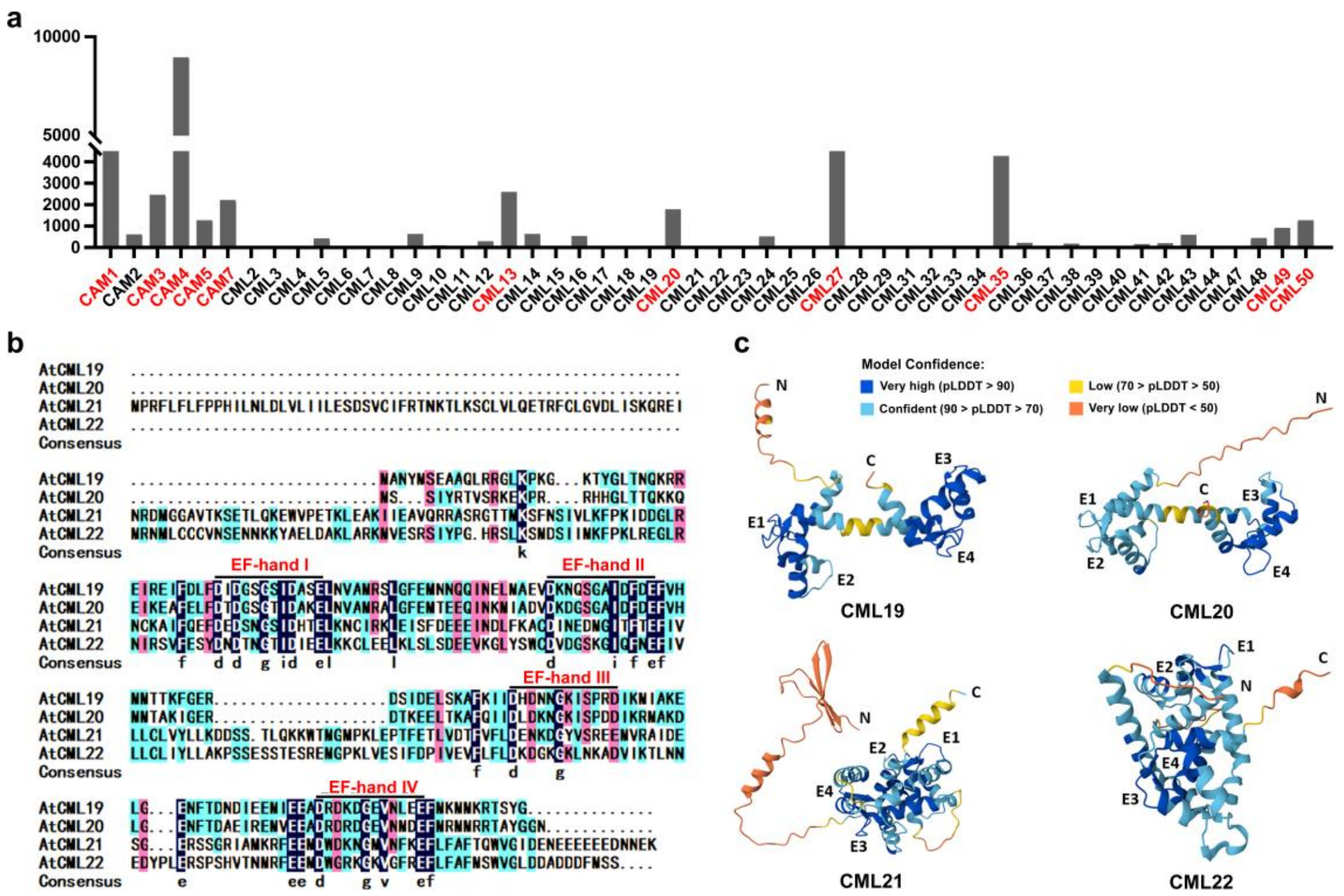

Supplementary Fig. 3. CML19/20/21/22 protein sequences alignment and predicted structures.

a Gene expression levels of *CAM*/*CML* genes in seeds, based on the microarray data from specific organs provided in McCormack et al. (2005). Genes with expression values >900 were selected as high-expression genes in seeds.

b Protein sequence alignment of CML19, CML20, CML21 and CML22 using DNAMAN. “Consensus” indicates residues identical across CML19/20/21/22. EF-hand motifs I–IV are indicated.

c AlphaFold-predicted structures of CML19, CML20, CML21 and CML22. pLDDT represents the per-residue confidence score. N and C denote the N- and C-termini. E1–E4 denote EF-hand domains.

Source data are provided in the Source Data file.

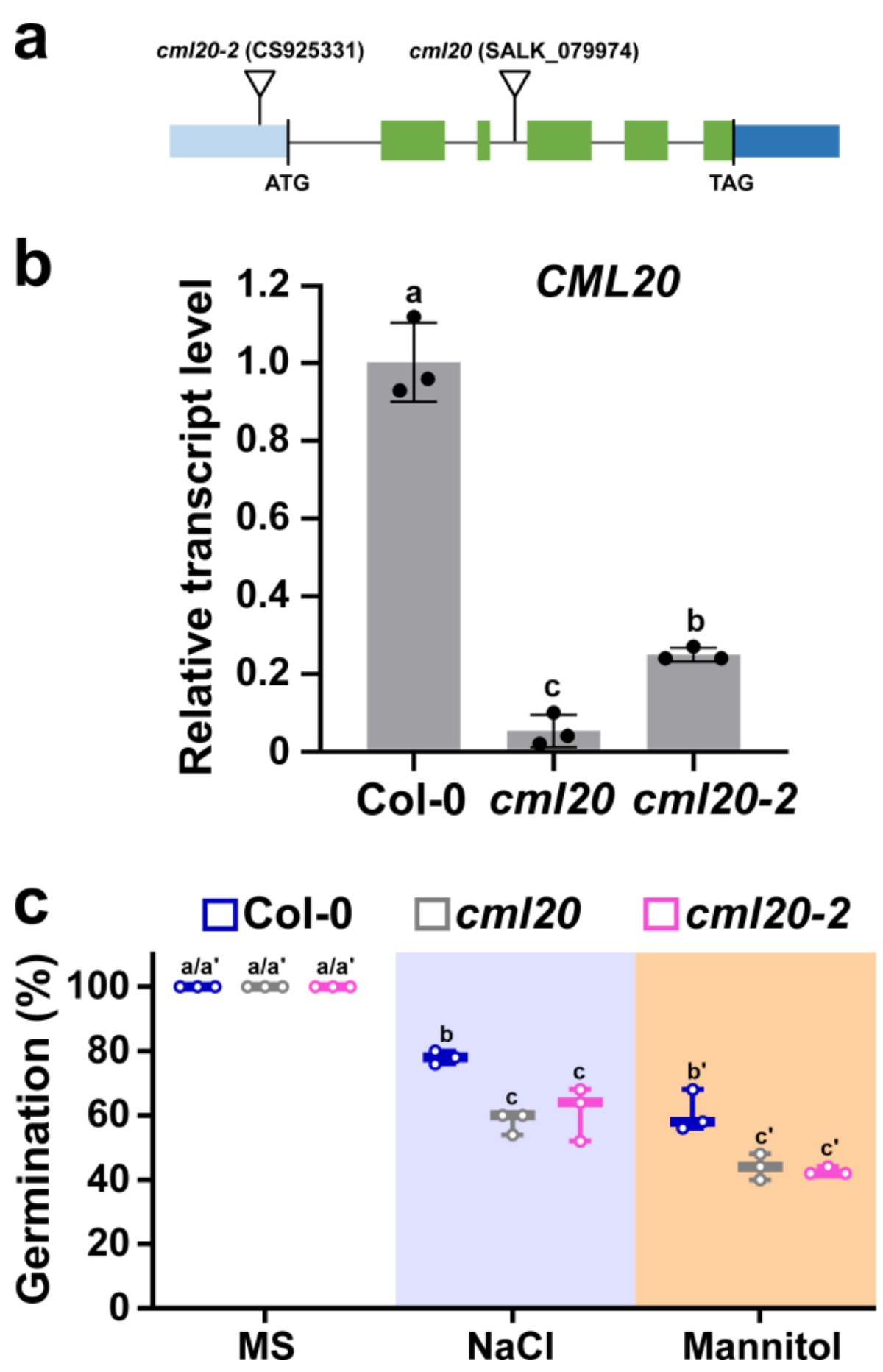

Supplementary Fig. 4. Expression of *CML20* and germination phenotypes of two T-DNA insertion *cml20* mutants under salt or osmotic stress.

a Schematic diagram showing the T-DNA insertion sites in the *cml20* (SALK_079974) and *cml20-2* (CS925331) alleles.

b RT-qPCR analysis of *CML20* transcript levels in Col-0, *cml20*, and *cml20-2*. *UBQ5* was used as the internal control for normalizing transcript levels. Data are means ± SD; $n = 3$ technical replicates. One-way ANOVA followed by Tukey's multiple comparisons test; different letters indicate significant differences ($p < 0.05$).

c Germination rates of Col-0, *cml20*, and *cml20-2* under salt and osmotic stress. Seeds were sown on MS medium containing 125 mM NaCl or 300 mM mannitol, stratified at 4 ℃ for 3 d in the dark, and incubated in continuous white light for 5 d. $n = 3$ biological replicates. Two-way ANOVA followed by Tukey's multiple comparisons test; different letters indicate significant differences ($p < 0.05$).

Source data and the exact *p* values are provided in the Source Data file.

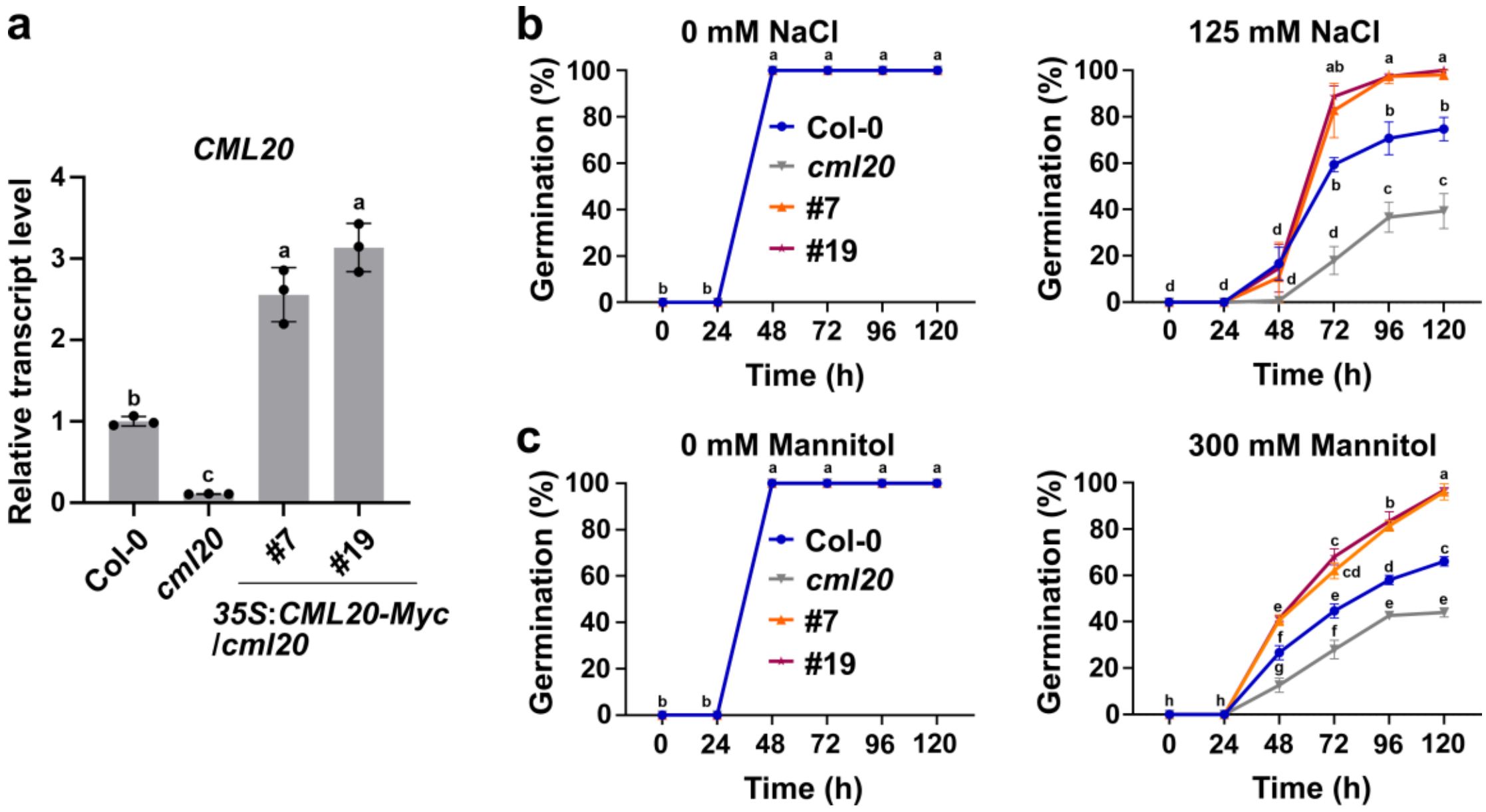

Supplementary Fig. 5. Expression of *CML20* and germination phenotypes of *CML20* complemented lines under salt or osmotic stress.

a RT-qPCR analysis of *CML20* expression in Col-0, *cml20*, and two complemented *35S:CML20-Myc*/*cml20* lines. *UBQ5* was used as the internal control for normalizing transcript levels. Data are means ± SD; $n = 3$ technical replicates. One-way ANOVA followed by Tukey's multiple comparisons test; different letters indicate significant differences ($p < 0.05$).

b, c Time-course germination of Col-0, *cml20*, and two *35S:CML20-Myc*/*cml20* lines under salt (**b**) or osmotic (**c**) stress. Seeds were sown on MS medium containing 125 mM NaCl (**b**) or 300 mM mannitol (**c**), stratified at 4 ℃ for 3 d in darkness, and incubated in continuous white light. Germination was recorded every 24 h. #7 and #19 are independent *35S:CML20-Myc*/*cml20* transgenic lines. Data are means ± SD; $n = 3$ biological replicates. Two-way ANOVA followed by Tukey's multiple comparisons test; different letters indicate significant differences ($p < 0.05$).

Source data and the exact *p* values are provided in the Source Data file.

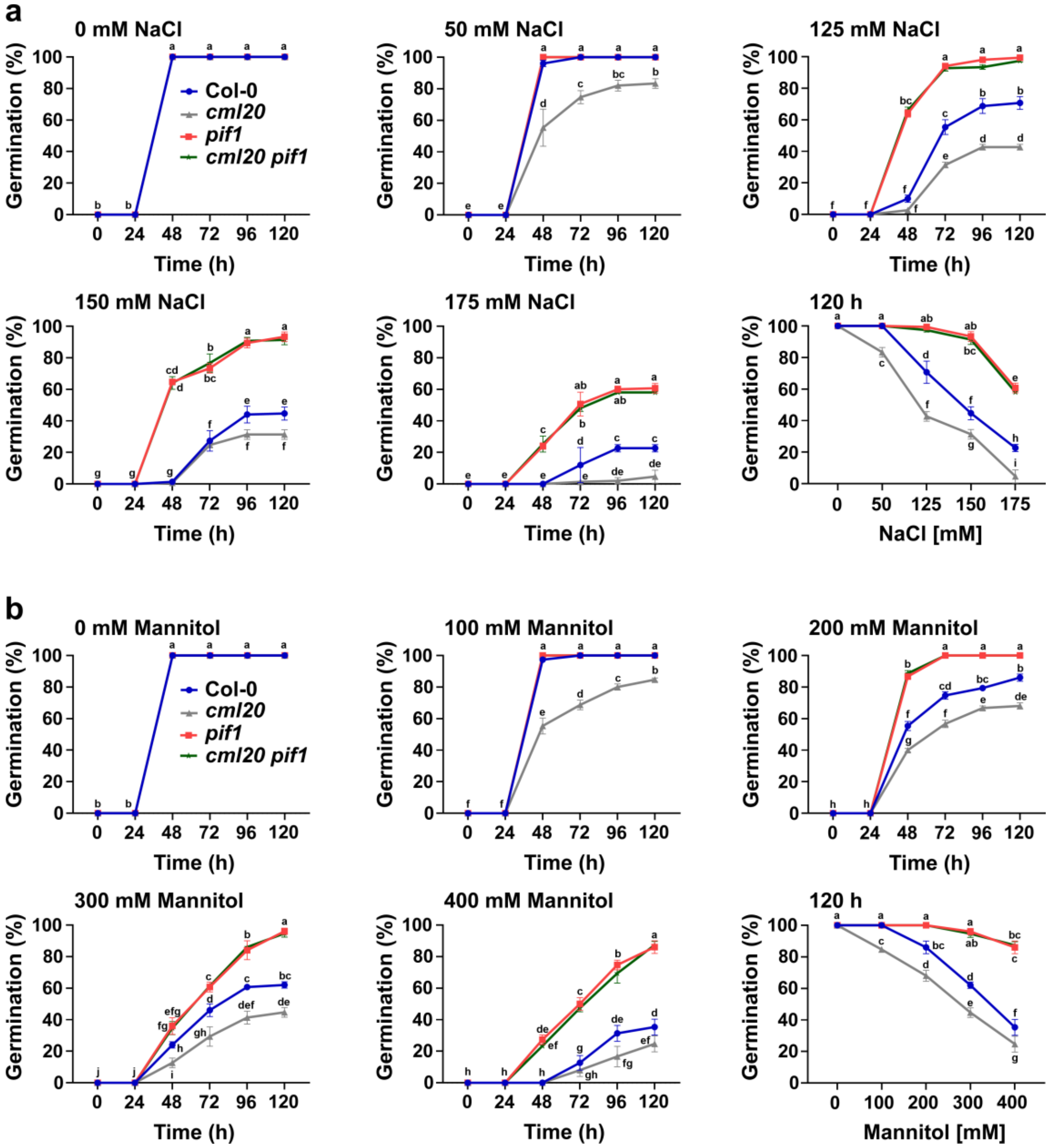

Supplementary Fig. 6. Germination time course of Col-0, *cml20*, *pif1* and *cml20 pif1* under salt or osmotic stress.

a, b Germination time course of Col-0, *cml20*, *pif1* and *cml20 pif1* seeds on MS medium with the indicated concentrations of NaCl (**a**) or mannitol (**b**). Seeds were sown on MS medium, stratified at 4 ℃ in darkness for 3 d, and then transferred to continuous white light. Germination was recorded every 24 h. Data are means ± SD; $n = 3$ biological replicates. Two-way ANOVA followed by Tukey’s multiple comparisons test; different letters indicate significant differences ($p < 0.05$). Source data and the exact p values are provided in the Source Data file.

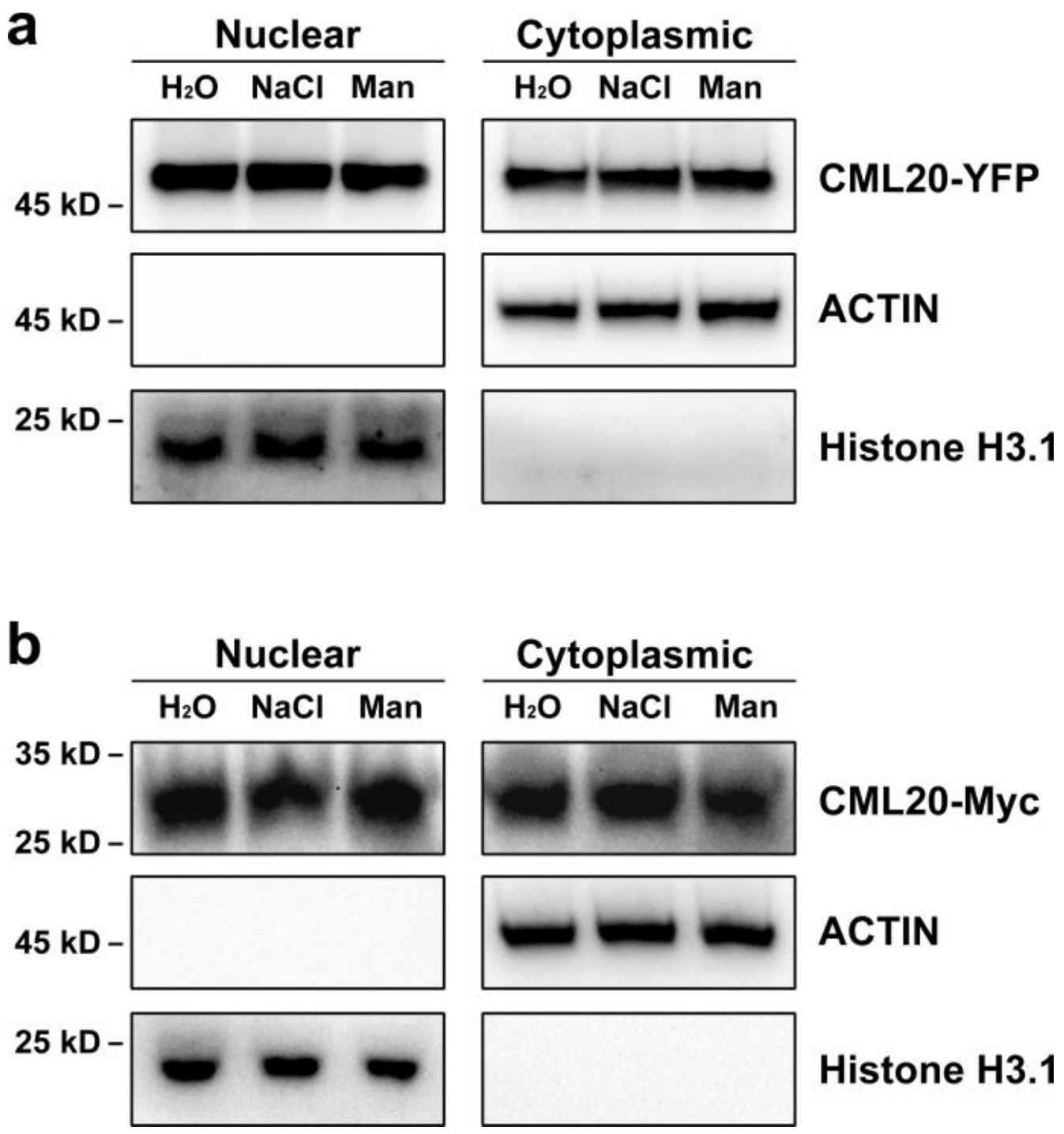

Supplementary Fig. 7. CML20 does not undergo stress-induced subcellular relocalization in imbibed seeds.

a, b Nuclear-cytoplasmic fractionation followed by Western blot analysis of CML20-YFP and CML20-Myc in *35S*:*CML20-YFP*/*cml20* (**a**) and *35S*:*CML20-Myc*/*cml20* (**b**) transgenic seeds. Seeds were imbibed in the dark for 24 h and then treated with 125 mM NaCl or 660 mM mannitol (Man) for 1 h. Histone H3.1 and ACTIN serve as markers for the nuclear and cytoplasmic fractions, respectively. Data are representative of two (**a**) or three (**b**) independent experiments with similar results. Source data are provided in the Source Data file.

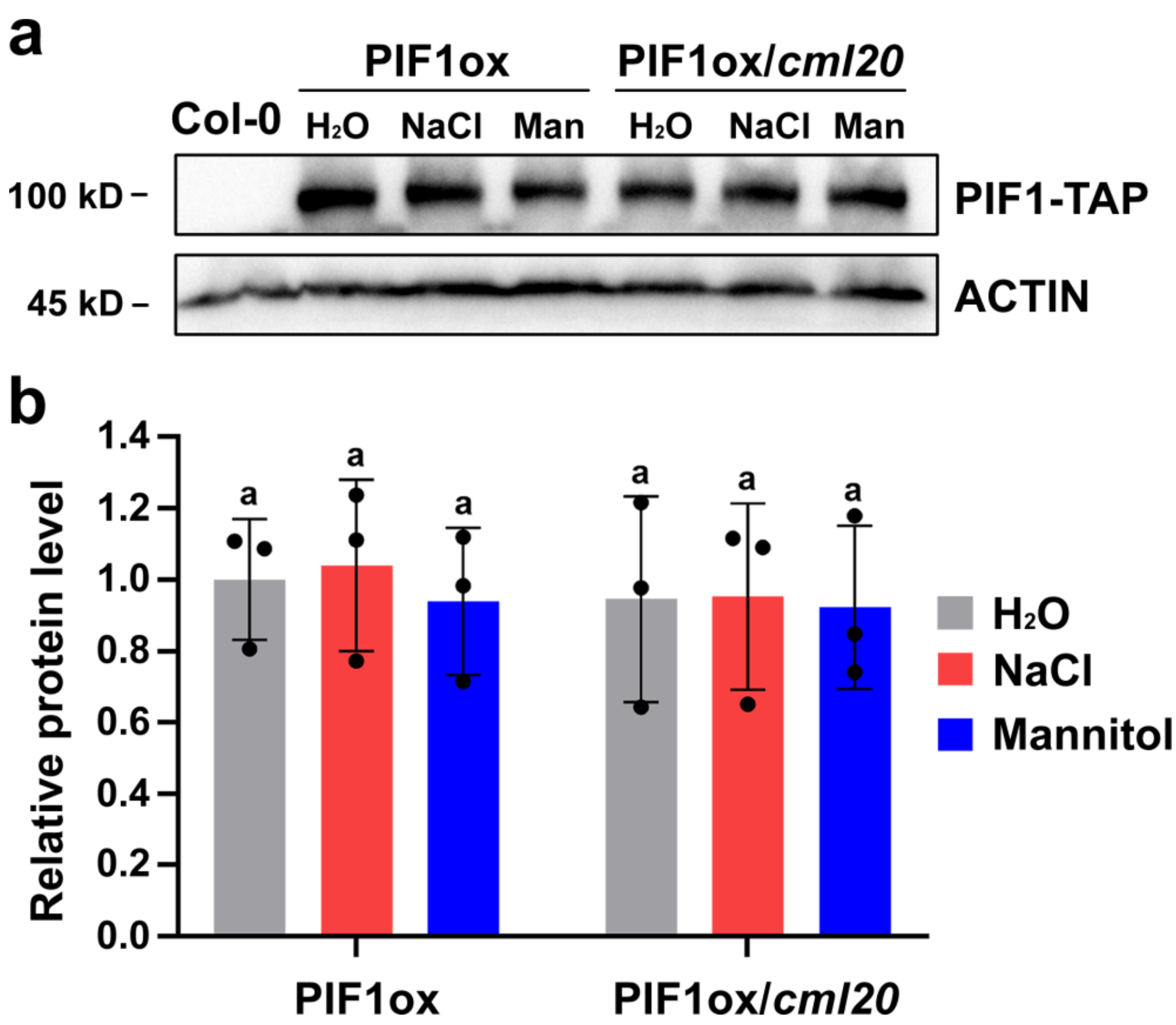

Supplementary Fig. 8. CML20 does not regulate PIF1 protein stability under control or stress conditions.

a, b Representative western blot images (**a**) and quantitative analysis (**b**) of PIF1-TAP protein levels in PIF1ox and PIF1ox/*cml20* seeds. Seeds were imbibed in the dark for 24 h and then treated for 1 h with H_2O (Control), 125 mM NaCl or 660 mM mannitol (Man). Col-0 was used as a negative control, and ACTIN served as a loading control. PIF1-TAP signals probed with anti-Myc antibody were normalized to ACTIN, with the H_2O-treated PIF1ox sample set to 1. Data are means ± SD; n = 3 biological replicates. Two-way ANOVA followed by Tukey's multiple comparisons test; different letters indicate significant differences (p < 0.05). Source data and the exact p values are provided in the Source Data file.

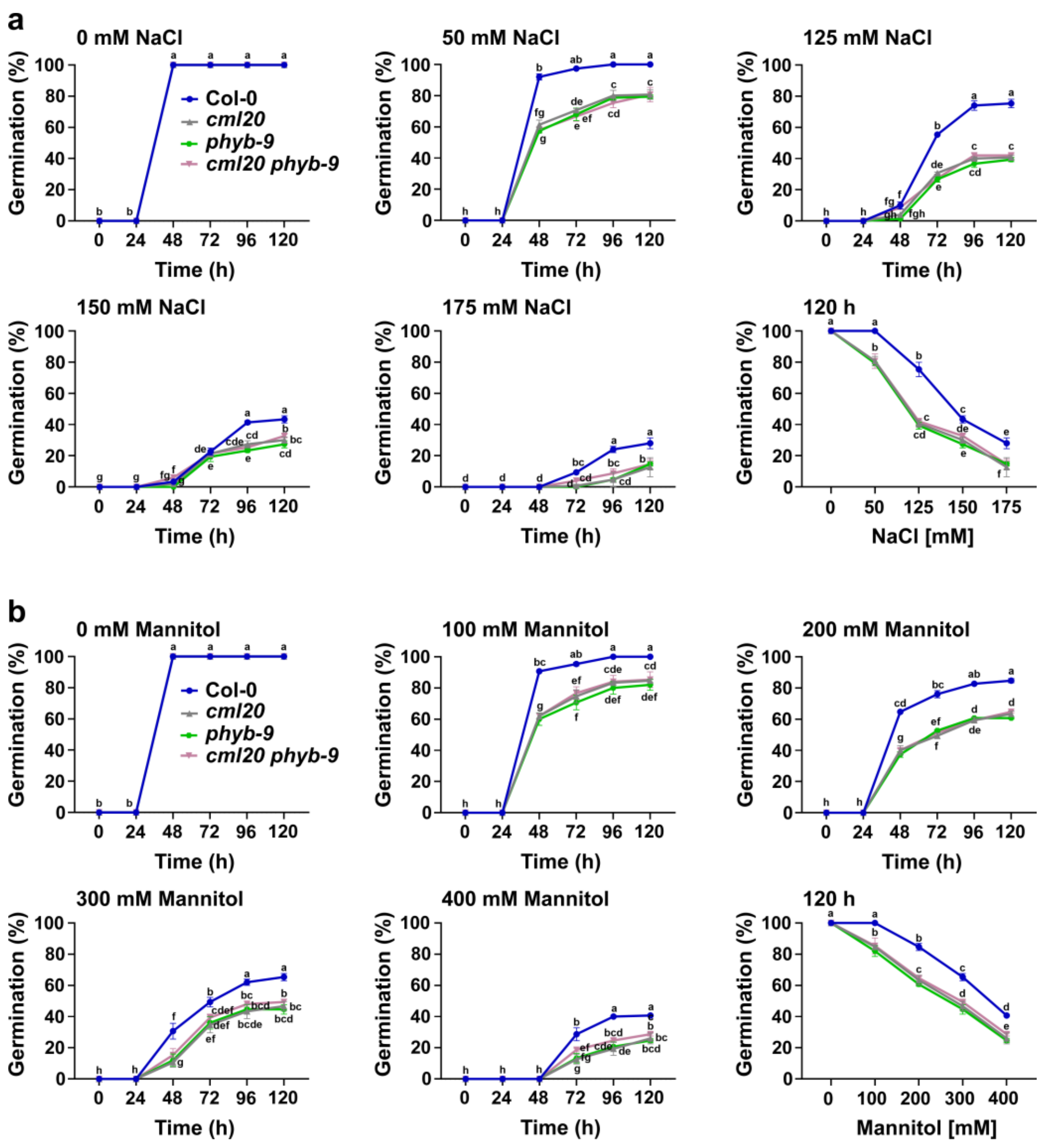

Supplementary Fig. 9. Germination time course of Col-0, *cml20, phyb-9*, and *cml20 phyb-9* under salt or osmotic stress.

a, b Germination time course of Col-0, *cml20*, *phyb-9*, and *cml20 phyb-9* seeds on MS medium with the indicated concentrations of NaCl (**a**) or mannitol (**b**). Seeds were sown on medium, stratified at 4 ℃ in darkness for 3 d, and then transferred to continuous white light. Germination was recorded every 24 h. Data are means ± SD; $n = 3$ biological replicates. Two-way ANOVA followed by Tukey's multiple comparisons test; different letters indicate significant differences ($p < 0.05$). Source data and the exact p values are provided in the Source Data file.

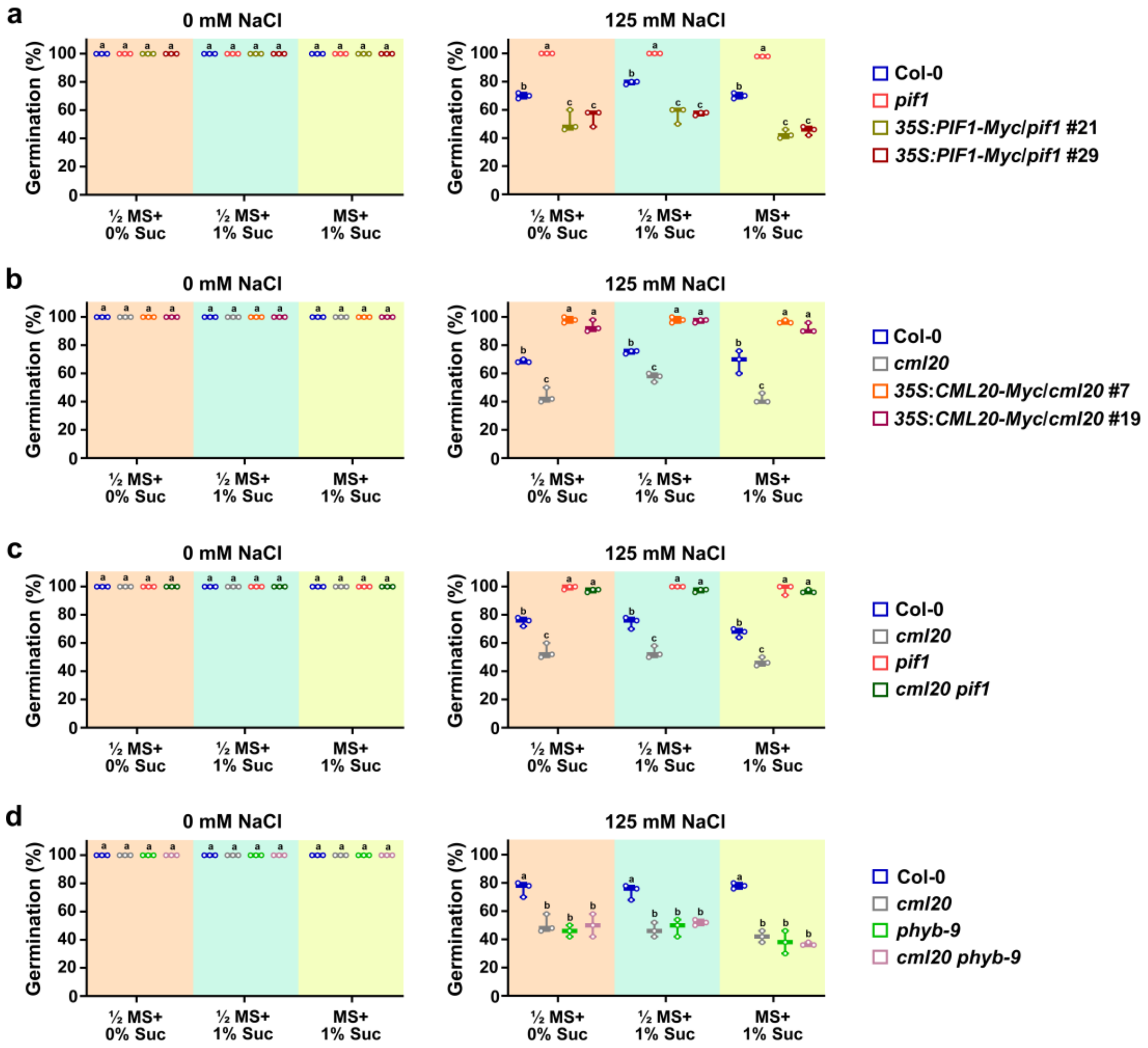

Supplementary Fig. 10. Key germination phenotypes under different media conditions in response to salt stress.

a–d Germination assays were performed on three media: half-strength MS without sucrose (½ MS+0% Suc), half-strength MS with 1% sucrose (½ MS+1% Suc), and full-strength MS with 1% sucrose (MS+1% Suc). After sowing, seeds were stratified at 4 ℃ in darkness for 3 d, and then incubated in continuous white light for 5 d. For each panel, germination is shown under control (0 mM NaCl, left) and salt stress (125 mM NaCl, right) conditions. Four genotype sets are presented: (**a**) Col-0, *pif1*, and two independent *35S:PIF1-Myc/pif1* lines; (**b**) Col-0, *cml20*, and two independent *35S:CML20-Myc/cml20* lines; (**c**) Col-0, *cml20*, *pif1*, and *cml20 pif1*; (**d**) Col-0, *cml20*, *phyb-9*, and *cml20 phyb-9*. $n = 3$ biological replicates. For each medium and treatment, one-way ANOVA followed by Tukey's multiple comparisons test was performed separately; different letters above the data points indicate significant differences among genotypes within the same medium ($p < 0.05$). Source data and the exact p values are provided in the Source Data file.

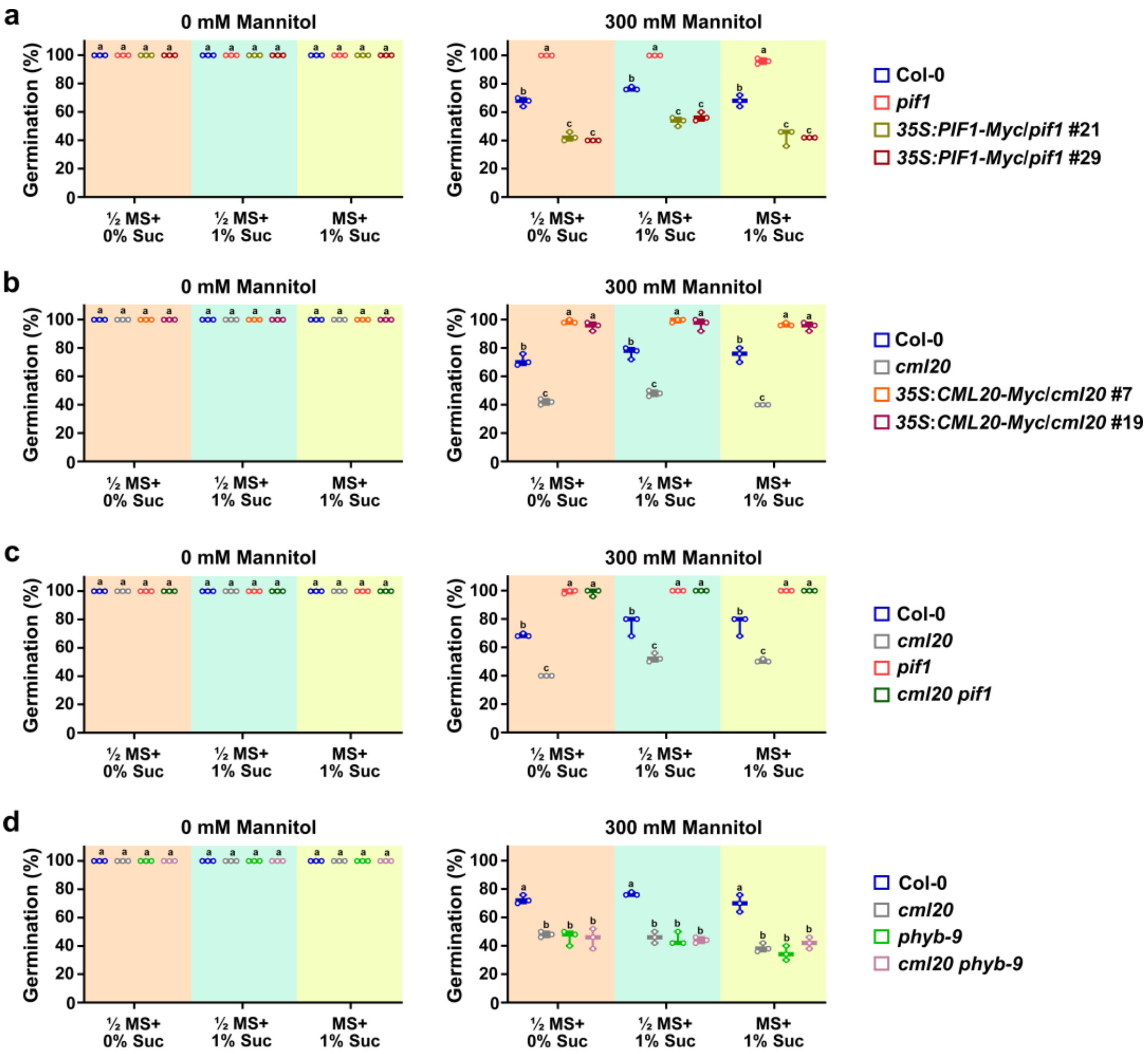

Supplementary Fig. 11. Key germination phenotypes under different media conditions in response to osmotic stress.

a–d Germination assays were performed on three media: half-strength MS without sucrose (½ MS+0% Suc), half-strength MS with 1% sucrose (½ MS+1% Suc), and full-strength MS with 1% sucrose (MS+1% Suc). After sowing, seeds were stratified at 4 ℃ in darkness for 3 d, and then incubated in continuous white light for 5 d. For each panel, germination is shown under control (0 mM mannitol, left) and osmotic stress (300 mM mannitol, right) conditions. Four genotype sets are presented: (**a**) Col-0, *pif1*, and two independent *35S:PIF1-Myc*/*pif1* lines; (**b**) Col-0, *cml20*, and two independent *35S:CML20-Myc*/*cml20* lines; (**c**) Col-0, *cml20*, *pif1*, and *cml20 pif1*; (**d**) Col-0, *cml20*, *phyb-9*, and *cml20 phyb-9*. $n = 3$ biological replicates. For each medium and treatment, one-way ANOVA followed by Tukey's multiple comparisons test was performed separately; different letters above the data points indicate significant differences among genotypes within the same medium ($p < 0.05$). Source data and the exact p values are provided in the Source Data file.

Supplementary Table 1. List of oligonucleotide sequences in this study.

**Plasmid construction**	
**Primer Name**	**Primer Sequence (5'→3')**
**pENTR4-CML20-F**	CGAGTCGACATGTCGAGTATATACAGAACTGTTT<br>CGAG
**pENTR4-CML20-R**	ATTTGCGGCCGCGAGTTACCACCATAAGCAGTTC<br>TCCT
**pENTR4-CML13-F**	CAATTCAGTCGACTGGATCCGGATGGGGAAAGA<br>TGGTCTGAGCG
**pENTR4-CML13-R**	CAAGAAAGCTGGGTCTAGATATCTCGAGTGCTTA<br>GCAACCATCCTTGCTATGAAATCTTC
**pENTR4-CML27-F**	CAATTCAGTCGACTGGATCCGGTACCGAATGGCA<br>AGCGCGAATCCAGAAAC
**pENTR4-CML27-R**	CAAGAAAGCTGGGTCTAGATATCTCGAGTGGGT<br>CGAAGGAGGAGCGGCGG
**pENTR4-CML35-F**	CAATTCAGTCGACTGGATCCGGTACCGAATGAA<br>GCTCGCCGCTAGCCT
**pENTR4-CML35-R**	CAAGAAAGCTGGGTCTAGATATCTCGAGTGATG<br>ATGATGATCATTCATCGCTGGAACC
**pENTR4-CML49-F**	CAATTCAGTCGACTGGATCCGGTACCGAATGTCT<br>GGTTATCCTCCATCAAGCCAAG
**pENTR4-CML49-R**	CAAGAAAGCTGGGTCTAGATATCTCGAGTGCAG<br>AAAACCCAAGGCTCATGAG
**pENTR4-CML50-F**	CAATTCAGTCGACTGGATCCGGTACCGAATGTCA<br>GGATATCCTCCGACTAGCC
**pENTR4-CML50-R**	CAAGAAAGCTGGGTCTAGATATCTCGAGTGTGC<br>GATGAGGAAGGGGAGGA
**pENTR4-CAM1-F**	CAATTCAGTCGACTGGATCCGGATGGCGGATCA<br>ACTCACTGAC
**pENTR4-CAM1-R**	CAAGAAAGCTGGGTCTAGATATCTCGAGTGCTTA<br>GCCATCATAATCTTGACAAACTC
**pENTR4-CAM3-F**	CAATTCAGTCGACTGGATCCGGATGGCGGATCA<br>GCTCACC
**pENTR4-CAM3-R**	CAAGAAAGCTGGGTCTAGATATCTCGAGTGCTTA<br>GCCATCATGACCTTAACAAACT
**pENTR4-CAM4-F**	CAATTCAGTCGACTGGATCCGGATGGCGGATCA<br>GCTAACTGAT
**pENTR4-CAM4-R**	CAAGAAAGCTGGGTCTAGATATCTCGAGTGCTTA<br>GCCATCATAATCTTGACAAACTCC

**pENTR4-CAM5-F**	CAATTCAGTCGACTGGATCCGGATGGCAGATCA GCTCACCG
**pENTR4-CAM5-R**	CAAGAAAGCTGGGTCTAGATATCTCGAGTGGAG AATACGGCAGTGACTTTTCC
**pENTR4-CAM7-F**	CAATTCAGTCGACTGGATCCGGATGGCGGATCA GCTAACCG
**pENTR4-CAM7-R**	CAAGAAAGCTGGGTCTAGATATCTCGAGTGTGCC ATCATGACTTTGACGAAC
**pGEX4T-CML20-F**	TGGATCCCCGGAATTCCCGGGTCGACTCATGTCG AGTATATACAGAACTGTTTCGAGAAA
**pGEX4T-CML20-R**	GCAGATCGTCAGTCAGTCACGATGCGGCCGCTCG TTACCACCATAAGCAGTTCTCCTCA
**pET28a-CML20-F**	AATTCGAGCTCCGTCGACAAATGTCGAGTATATA CAGAACTGTTTCGAGAAA
**pET28a-CML20-R**	TGGTGGTGCTCGAGTGCGGCCGCGTTACCACCAT AAGCAGTTCTCCTCA
**pET28a-CML20-mt-F**	AATTCGAGCTCCGTCGACAAATGTCGAGTATATA CAGAACTGTTTCGAGAAA
**pET28a-CML20-mt-R**	TGGTGGTGCTCGAGTGCGGCCGCGTTACCACCAT AAGCAGTTCTCCTCA
**pGWB-435-ABI5p-F**	AGTCGACTGGATCCGGTACCGCATTTAAAATTCG ATAGTCCTTATGCA
**pGWB-435-ABI5p-R**	TGTACAAGAAAGCTGGGTCTAGATATCTTAACAA CTGCATCATATACACAACAATAA
**Genotyping**	
**Primer Name**	**Primer Sequence (5’→3’)**
**pif1-LP**	CTCTTTGGATCTTTCTGGGG
**pif1-RP**	GACTTGCGCACGATAGCTAAC
**cml20-LP**	GATGTAAGCTTTCAAAGGTCGG
**cml20-RP**	CATCATGGATTGACGACACAG
**cml20-2-LP**	TTCCATCTTTATCCACATCAGC
**cml20-2-RP**	TGACTTTTGTCCATGGGAGAG
**LB1.3**	TAGCATCTGAATTTCATAACCAATCTCGATACAC

**EMSA**	
**Primer Name**	**Primer Sequence (5’→3’)**
**ABI5probe-F-biotin**	ACAGAAGAGAGACACGTGCAGGACACGTGTCGTCCGCAGCC
**ABI5probe-R-biotin**	GGCTGCGGACGACACGTGTCCTGCACGTGTCTCTCTTCTGT
**ChIP-qPCR**	
**Primer Name**	**Primer Sequence (5’→3’)**
**ChIP-PIL1p-F**	CACAAAGGGGTGGATGAATCACG
**ChIP-PIL1p-R**	ACTGTAGTGAGTGACATGCGAGAG
**ChIP-PIL2p-F**	AGGACAAGTGTCTAATACACTGCATACG
**ChIP-PIL2p-R**	ACGTGGTTGTGGGAAGAACATTCA
**ChIP-SOMp-F**	GGTAGAAACCAAAAATGTATTTGCTTCCATC
**ChIP-SOMp-R**	ACAACACAGAAAGGATATGCACGAGATTAAAT
**ChIP-ABI5p-F**	GAGCCTGTGAGAAGACACTG
**ChIP-ABI5p-R**	GTCTAAAGTTATCTTTCGACCAATGGA
**ChIP-GA3ox1p-F**	CAAACGATCAATCGTCTACTTTGC
**ChIP-GA3ox1p-R**	CACCAACGTTCCCTAAGATTTTCA
**RT-qPCR**	
**Primer Name**	**Primer Sequence (5’→3’)**
**RT-PIF1-F**	TGAATCCCGTAGCGAGGAAACAA
**RT-PIF1-R**	TTCCACATCCCATTGACATCATCTG
**RT-ACTIN2-F**	TCCCTCAGCACATTCCAGCA

**RT-ACTIN2-R**	GATCCCATTCATAAAACCCCAGC
**RT-CML19-F**	ACAACCAAATTCGGAGAACGAGAC
**RT-CML19-R**	CTTCTCCATCTTTGTCACGGTCTG
**RT-CML20-F**	CGCATGGCAAAGGACTTGGGT
**RT-CML20-R**	CCATCACGGTCTCGGTCTGCT
**RT-CML21-F**	GAGGAGATGGTCCGAGCCATTGA
**RT-CML21-R**	GTCCCAGTCCATTTCCTCAAATCTC
**RT-CML22-F**	GACATTGAACAACGAAGACTATCCGC
**RT-CML22-R**	TCCCCAATCCATTTCTTCAAATCTCA
**RT-UBQ5-F**	ACCACTTCGACCGCCACTACT
**RT-UBQ5-R**	ACGCCTAAGCCTGCTGGTT
