# Supplementary material for: CML20 integrates phytochrome and calcium signaling pathways to optimize seed germination under abiotic stress
Source: Nat Commun. 2026 Sep 4;17:10540. doi: 10.1038/s41467-026-77567-0 (PMC13642242; doi:10.1038/s41467-026-77567-0)
Supplement: Supplementary file 2 [file 41467_2026_77567_MOESM2_ESM.pdf]

nature portfolio

Corresponding author(s): Hui Shi

Last updated by author(s): Aug 19, 2026

Reporting Summary

Nature Portfolio wishes to improve the reproducibility of the work that we publish. This form provides structure for consistency and transparency in reporting. For further information on Nature Portfolio policies, see our Editorial Policies and the Editorial Policy Checklist.

Statistics

For all statistical analyses, confirm that the following items are present in the figure legend, table legend, main text, or Methods section.

n/a	Confirmed	
☐	☒	The exact sample size (*n*) for each experimental group/condition, given as a discrete number and unit of measurement
☐	☒	A statement on whether measurements were taken from distinct samples or whether the same sample was measured repeatedly
☐	☒	The statistical test(s) used AND whether they are one- or two-sided<br>*Only common tests should be described solely by name; describe more complex techniques in the Methods section.*
☒	☐	A description of all covariates tested
☐	☒	A description of any assumptions or corrections, such as tests of normality and adjustment for multiple comparisons
☐	☒	A full description of the statistical parameters including central tendency (e.g. means) or other basic estimates (e.g. regression coefficient) AND variation (e.g. standard deviation) or associated estimates of uncertainty (e.g. confidence intervals)
☐	☒	For null hypothesis testing, the test statistic (e.g. *F*, *t*, *r*) with confidence intervals, effect sizes, degrees of freedom and *P* value noted<br>*Give P values as exact values whenever suitable.*
☒	☐	For Bayesian analysis, information on the choice of priors and Markov chain Monte Carlo settings
☒	☐	For hierarchical and complex designs, identification of the appropriate level for tests and full reporting of outcomes
☒	☐	Estimates of effect sizes (e.g. Cohen's *d*, Pearson's *r*), indicating how they were calculated

Our web collection on statistics for biologists contains articles on many of the points above.

Software and code

Policy information about availability of computer code

Data collection	Plant phenotypes were imaged using a stereomicroscope (Leica M25). qPCR data were collected using an ABI Q6 Pro system. Confocal fluorescence images were acquired using a ZEISS LSM 780 confocal microscope. LUC signals were detected using a ChampChemi 610 Plus system (SageCreation). Calcium signals were recorded using a Berthold LB985 luminescence imager. Western blot and EMSA signals were detected using a Bio-Rad imaging system.
Data analysis	Statistical analyses, including one-way ANOVA, two-way ANOVA and t-test where applicable, were performed using GraphPad Prism version 8.4.2 (GraphPad Software, San Diego, CA, USA). Image quantification and intensity measurements were conducted using ImageJ. Protein sequence alignment and analysis were performed using DNAMAN. Protein structure prediction was performed using AlphaFold3 and visualized in Chimera X software.

For manuscripts utilizing custom algorithms or software that are central to the research but not yet described in published literature, software must be made available to editors and reviewers. We strongly encourage code deposition in a community repository (e.g. GitHub). See the Nature Portfolio guidelines for submitting code & software for further information.

Data

Policy information about availability of data

All manuscripts must include a data availability statement. This statement should provide the following information, where applicable:

- Accession codes, unique identifiers, or web links for publicly available datasets
- A description of any restrictions on data availability
- For clinical datasets or third party data, please ensure that the statement adheres to our policy

All data supporting the findings of this study are available within the paper and Supplementary Information. Source data are provided with this paper.

Research involving human participants, their data, or biological material

Policy information about studies with human participants or human data. See also policy information about sex, gender (identity/presentation), and sexual orientation and race, ethnicity and racism.

Reporting on sex and gender	not applicable
Reporting on race, ethnicity, or other socially relevant groupings	not applicable
Population characteristics	not applicable
Recruitment	not applicable
Ethics oversight	not applicable

Note that full information on the approval of the study protocol must also be provided in the manuscript.

Field-specific reporting

Please select the one below that is the best fit for your research. If you are not sure, read the appropriate sections before making your selection.

☒ Life sciences ☐ Behavioural & social sciences ☐ Ecological, evolutionary & environmental sciences

For a reference copy of the document with all sections, see nature.com/documents/nr-reporting-summary-flat.pdf

Life sciences study design

All studies must disclose on these points even when the disclosure is negative.

Sample size	No statistical methods were used to predetermine sample sizes. The sample size for statistic analysis of each experiment was clearly described in the paper.
Data exclusions	No data were excluded from the analyses.
Replication	Replication statement is included in the figure legends.
Randomization	The experiments were not randomized.
Blinding	The investigators were not blinded to allocation during experiments and outcome assessment.

Reporting for specific materials, systems and methods

We require information from authors about some types of materials, experimental systems and methods used in many studies. Here, indicate whether each material, system or method listed is relevant to your study. If you are not sure if a list item applies to your research, read the appropriate section before selecting a response.

Materials & experimental systems

n/a	Involved in the study	
☐	☒	Antibodies
☐	☒	Eukaryotic cell lines
☒	☐	Palaeontology and archaeology
☒	☐	Animals and other organisms
☒	☐	Clinical data
☒	☐	Dual use research of concern
☐	☒	Plants

Methods

n/a	Involved in the study	
☒	☐	ChIP-seq
☒	☐	Flow cytometry
☒	☐	MRI-based neuroimaging

Antibodies

Antibodies used	anti-GFP (Abcam, ab13970, 1:2000),<br>anti-Myc (Sigma-Aldrich, M4439, 1:1000),<br>anti-ACTIN (Sigma-Aldrich, A0480, 1:50,000),<br>anti-RPN6 (Abmart, PA4990, 1:8000),<br>anti-MBP (NEB, E8032, 1:2000),<br>anti-His (ABclonal, AE086, 1:2000),<br>anti-HA (Cell Signaling Technology, 45346, 1:1000),<br>anti-Histone H3.1 (Abmart, 30266, 1:6000)<br>anti-GST (ABclonal, AE006, 1:3000).
Validation	Commercial antibodies were validated by the manufacturers for the indicated applications.

Eukaryotic cell lines

Policy information about cell lines and Sex and Gender in Research

Cell line source(s)	Human embryo kidney cells (HEK293T) (Procell, CL-0005)
Authentication	The cell line was authenticated by morphological inspection.
Mycoplasma contamination	The cells were tested negative for mycoplasma contamination.
Commonly misidentified lines (See ICLAC register)	No commonly misidentified cell lines were used in this study.

Dual use research of concern

Policy information about dual use research of concern

Hazards

Could the accidental, deliberate or reckless misuse of agents or technologies generated in the work, or the application of information presented in the manuscript, pose a threat to:

No	Yes	
☒	☐	Public health
☒	☐	National security
☒	☐	Crops and/or livestock
☒	☐	Ecosystems
☒	☐	Any other significant area

Experiments of concern

Does the work involve any of these experiments of concern:

No	Yes	
☒	☐	Demonstrate how to render a vaccine ineffective
☒	☐	Confer resistance to therapeutically useful antibiotics or antiviral agents
☒	☐	Enhance the virulence of a pathogen or render a nonpathogen virulent
☒	☐	Increase transmissibility of a pathogen
☒	☐	Alter the host range of a pathogen
☒	☐	Enable evasion of diagnostic/detection modalities
☒	☐	Enable the weaponization of a biological agent or toxin
☒	☐	Any other potentially harmful combination of experiments and agents

Plants

Seed stocks	The information on the genotypes of the plants used in this study is listed in the Methods section.
Novel plant genotypes	This information is provided in the Methods section.
Authentication	This information is provided in the Methods section.
