# Supplementary material for: CML20 integrates phytochrome and calcium signaling pathways to optimize seed germination under abiotic stress
Source: Nat Commun. 2026 Sep 4;17:10540. doi: 10.1038/s41467-026-77567-0 (PMC13642242; doi:10.1038/s41467-026-77567-0)
Supplement: Supplementary file 3 [file 41467_2026_77567_MOESM3_ESM.pdf]

nature portfolio

Peer Review File

CML20 integrates phytochrome and calcium signaling pathways to optimize seed germination under abiotic stress

Corresponding Author: Professor Hui Shi

This file contains all reviewer reports in order by version, followed by all author rebuttals in order by version.

Version 0:

Reviewer comments:

Reviewer #1

(Remarks to the Author)
Summary
This study systematically investigates the role of CML20 in integrating light and calcium signaling during seed germination under salt and osmotic stress. The authors show that CML20 physically interacts with PIF1 in a Calcium-dependent manner and prevents its DNA binding activity in vitro and in vivo. CML20 also interacts with phyB in a light-independent manner and forms a ternary complex. By combining genetic, biochemical, and molecular approaches, the authors propose a phyB–CML20–PIF1 regulatory module that provides new mechanistic insight into how environmental cues are coordinated to optimize seed germination. Overall, the data are solid and the conclusions are largely well supported.

Major comments / Suggestions
1. In Figures 1A and 1B, the authors performed WT seed germination assays under both light and dark conditions. Fig. 1A/B show WT seeds germinate 100% under dark conditions with 0% NaCl or mannitol. This might be a mistake as WT seeds don't germinate without light stimulus. Moreover, only phyb-9 data under light conditions are shown. It would strengthen the comparison to also include the phyb-9 dark-grown samples under the same experimental conditions.
2. In lines 109–110, the authors state that pif3, pif4, and pif5 mutants are "indistinguishable from WT across all NaCl concentrations tested." Based on Figure 1C, there still appears to be a modest difference between WT and the pif4 mutant. This sentence should be rephrased to more accurately reflect the data.
3. In Figure 2B, CML expression was analyzed using mature seeds, whereas the relevant phenotype concerns seed germination. If possible, it would be more appropriate to examine CML expression in imbibed seeds during germination to better correlate gene expression with the observed phenotypes.
4. The authors show interactions between CML20 with PIF1 and also phyB. Are these interactions affected under salt/osmotic conditions?
5. In the proposed model, CML20 appears to bind Ca^{2+} under salt/osmotic stress and subsequently function in the nucleus, conceptually resembling the dynamic behavior of phyB. It would be helpful if the authors could clarify whether CML20 undergoes any subcellular localization changes between normal and salt/osmotic stress conditions, either experimentally or through additional discussion.
6. Fig. 8A and B, last column shows stronger interaction. However, 8A appears to show two distinct bands and 8B shows a faster migrating band. Are these degradation products or cross-reacting bands migrating closely?
7. Fig 2H/I and 8D/E, authors have performed epistasis analysis using a single concentration of salt/mannitol. It is better to perform using a concentration gradient to assess if phyB and pif1 are epistatic to cml20 under a broad range of stress condition.
8. It is not clear what light condition they are using when assaying for salt/osmotic effects on seed germination. Please clarify in the figure legends.

Reviewer #2

(Remarks to the Author)
I co-reviewed this manuscript with one of the reviewers who provided the listed reports. This is part of the Nature Communications initiative to facilitate training in peer review and to provide appropriate recognition for Early Career Researchers who co-review manuscripts.

Reviewer #3

(Remarks to the Author)
This study identified the calmodulin-like protein CML20 as a key molecular node connecting light signaling (phyB-PIF1) with abiotic stress signaling (Ca^{2+}), revealing a new mechanism through which the inhibition of seed germination by PIF1 is rapidly relieved in a non-proteolytic manner under stress conditions. This discovery holds significant theoretical importance for understanding how plants balance germination timing in unfavorable environments and also demonstrates potential agricultural application value. However, several issues still need to be addressed before publication.

1. This study starts from the phenomenon of the phyB-PIF1 module responding to stress to regulate seed germination and directly transitions to Ca^{2+}signaling. It lacks in planta evidence showing that salt/osmotic stress can indeed induce a cytosolic Ca^{2+}increase sufficient to activate CML20 during the early stage of seed imbibition.

2. This study indicates that the interaction between CML20 and PIF1 is Ca^{2+}-dependent (Fig. 4) and that CML20 is induced by stress (Figs. 2C-D), proposing that stress induces $Ca^{2+}$to promote the CML20-PIF1 interaction. However, evidence demonstrating the effect of stress on the CML20-PIF1 interaction is lacking.

3. This study states that CML20 does not affect PIF1 protein abundance, but it remains unclear whether PIF1 protein abundance is affected under stress conditions. This point is crucial for an accurate description of the model in fig. 9.

4. Figure 8 shows that phyB-Pfr can promote the CML20-PIF1 interaction, but the binding of phyB itself to CML20 is independent of light or Ca^{2+} (Fig. 7). This finding is interesting, but a mechanistic explanation is needed: Does phyB increase the encounter frequency between CML20 and PIF1, or does it induce a conformational change in PIF1 making it more recognizable by CML20? Some discussion on this is necessary.

5. Furthermore, as illustrated in the model, light-activated PHYB leads to the degradation of PIF1 protein. The authors claim that PHYB promotes the CML20-PIF1 interaction. It is required to elucidate how PHYB promotes the CML20-PIF1 protein interaction while simultaneously promoting PIF1 degradation.

6.The in vitro pull-down assay used 0.5 mM $CaCl_2$ (Methods section), which is far higher than physiological cytosolic calcium peaks (typically 0.1-10 μM). Please explain the rationale for this concentration, or supplement with dose-response experiments within the physiological concentration range to verify physiological relevance.

7.The Arabidopsis phyB-9 mutant carries an additional mutation in the VENOSA4 gene (Plant Physiol. 2018 Sep;178(1):3-6). Therefore, to confirm that the observed germination phenotype under stress conditions is specifically attributable to phyB loss of function, the authors should include other phyB mutant alleles in their analysis.

Reviewer #4

(Remarks to the Author)
This manuscript presents a comprehensive study addressing how light and calcium signaling pathways are integrated to regulate seed germination under abiotic stress conditions. The authors identify CALMODULIN-LIKE 20 (CML20) as a key calcium sensor that promotes germination tolerance to salt and osmotic stress, and demonstrate that CML20 directly interacts with the transcription factor PIF1 in a Ca^{2+}-dependent manner, thereby suppressing PIF1 self-association and DNA-binding activity through a non-proteolytic mechanism. Furthermore, the study shows that phytochrome B (phyB) independently associates with both CML20 and PIF1, facilitating the formation of a CML20–PIF1 complex and further attenuating PIF1 transcriptional activity. Genetic analyses place PIF1 downstream of CML20 and indicate that CML20 and phyB function within the same signaling pathway. Importantly, the work integrates a broad range of genetic, molecular, biochemical, and physiological approaches, which together provide strong and coherent support for the proposed phyB–CML20–PIF1 signaling module.
Major comments
A central pillar of the study relies on the phenotypic and molecular characterization of the cml20 mutant derived from the T-DNA insertion line SALK_079974. While the data obtained using this line are internally consistent, conclusions drawn from a single mutant allele may be confounded by allele-specific effects, secondary mutations, or positional effects associated with T-DNA insertions. Therefore, independent validation using additional cml20 alleles—such as other insertion lines or CRISPR-generated loss-of-function mutants—is essential to robustly attribute the observed phenotypes to CML20 function and to strengthen the generality and reliability of the proposed model.
A major concern regarding the germination assays is the use of exogenous sucrose, which is known to promote seed germination by providing an external carbon source and thereby alleviating the requirement for early establishment of photosynthetic activity. As a result, sucrose can partially bypass developmental and energetic constraints that are normally imposed during germination under stress conditions, including salinity and altered light regimes. In addition to its well-documented role as a signaling molecule, sucrose therefore fundamentally alters the physiological context of germination, making it difficult to attribute the observed phenotypes specifically to salt stress or light–phytochrome signaling. For this reason, germination assays aimed at dissecting salt and light responses are typically performed in the absence of sucrose, often on reduced-strength (½×) MS medium to minimize additional osmotic or nutritional effects. The authors should justify the inclusion of sucrose or confirm that the key conclusions hold under sugar-free conditions.
The study assumes that salt/osmotic stress generates a Ca^{2+} signal relevant to CML20; however, no direct measurement of Ca^{2+} dynamics in seeds is provided. It is recommended to include, at minimum, an explicit discussion of this point, or a reference to relevant work in seeds. Alternatively, an experiment using a Ca^{2+} chelator (e.g., EGTA or BAPTA) during

germination could strengthen the functional link between stress, Ca^{2+} signaling, and CML20 activity.
Minor comment:
For clarity and consistency, it would be preferable to label the y-axis as "Relative transcript level" (e.g., Relative CML20 transcript level), which more accurately reflects the normalized RT-qPCR-based expression data.

Version 1:

Reviewer comments:

Reviewer #1

(Remarks to the Author)
The authors have adequately addressed most of my concerns except they did not examine the subcellular localization of CML20. They have CML20-GFP transgenic plants in PIF1OX background. This can be done easily to examine where the protein is located before and after stress conditions. This can be very helpful in the context of this story.

Reviewer #2

(Remarks to the Author)
I co-reviewed this manuscript with one of the reviewers who provided the listed reports. This is part of the Nature Communications initiative to facilitate training in peer review and to provide appropriate recognition for Early Career Researchers who co-review manuscripts.

Reviewer #3

(Remarks to the Author)
The author has fully addressed my concerns in the revised version. I have no further worries, and I believe the current version is suitable for publication.

Reviewer #4

(Remarks to the Author)
I would like to commend the authors for their thorough and thoughtful responses to the reviewers' comments; their efforts to address the feedback and improve the manuscript are highly appreciated. There is, however, just one remaining point that requires further attention before publication.
The authors' effort to provide a complementation line for the cml20 mutant is highly valued and indeed confirms that the observed phenotype is linked to the functional loss of this locus. However, relying on a single T-DNA insertion line remains a concern.
While complementation rescues the phenotype, it cannot rule out allele-specific artifacts. For instance, depending on the exact insertion site, a single T-DNA mutant can sometimes produce truncated transcripts with dominant-negative effects, or cause unintended neighboring gene disruption (position effects) that could be coincidentally altered during tissue culture or transformation. Furthermore, passenger mutations linked to this specific line cannot be entirely excluded.
Characterizing two independent mutant alleles is the standard benchmark in plant genetics to robustly confirm a causal link between a gene and a phenotype. Therefore, while a full characterization might not be necessary given that the complementation is clean, the authors should at least provide basic phenotypic data for a second independent cml20 allele to conclusively solidify their findings.

Open Access This Peer Review File is licensed under a Creative Commons Attribution 4.0 International License, which permits use, sharing, adaptation, distribution and reproduction in any medium or format, as long as you give appropriate credit to the original author(s) and the source, provide a link to the Creative Commons license, and indicate if changes were made.
In cases where reviewers are anonymous, credit should be given to 'Anonymous Referee' and the source.
The images or other third party material in this Peer Review File are included in the article's Creative Commons license, unless indicated otherwise in a credit line to the material. If material is not included in the article's Creative Commons license and your intended use is not permitted by statutory regulation or exceeds the permitted use, you will need to obtain permission directly from the copyright holder.
To view a copy of this license, visit https://creativecommons.org/licenses/by/4.0/

MS ID#: NCOMMS-25-93787

MS TITLE: CML20 integrates phytochrome and calcium signaling pathways to optimize seed germination under abiotic stress

Reviewer #1:

Summary

This study systematically investigates the role of CML20 in integrating light and calcium signaling during seed germination under salt and osmotic stress. The authors show that CML20 physically interacts with PIF1 in a Calcium-dependent manner and prevents its DNA binding activity in vitro and in vivo. CML20 also interacts with phyB in a light-independent manner and forms a ternary complex. By combining genetic, biochemical, and molecular approaches, the authors propose a phyB–CML20–PIF1 regulatory module that provides new mechanistic insight into how environmental cues are coordinated to optimize seed germination. Overall, the data are solid and the conclusions are largely well supported.

Major comments / Suggestions

1. In Figures 1A and 1B, the authors performed WT seed germination assays under both light and dark conditions. Fig. 1A/B show WT seeds germinate 100% under dark conditions with 0% NaCl or mannitol. This might be a mistake as WT seeds don't germinate without light stimulus. Moreover, only phyb-9 data under light conditions are shown. It would strengthen the comparison to also include the phyb-9 dark-grown samples under the same experimental conditions.

Our Reply:

We thank the reviewer for raising this important point regarding the light conditions in our germination assays. The reviewer is correct that Arabidopsis seeds typically require a light stimulus to germinate. In our experiments, seeds assigned to the "Dark" condition were exposed to white light to trigger germination and then incubated in darkness, as described in previous studies on germination under stress conditions (Ma et al., 2023; Ren et al., 2010; Yu et al., 2017). Detailed procedures are provided in the figure legend in the revised manuscript. To address this point unambiguously, we have performed two complementary experimental approaches.

(1) We repeated the assays under continuous white light (Light) or darkness (Dark), now including *phyb-9* in both conditions (revised Fig. 1A, B). On MS control medium, both WT and *phyb-9* seeds germinated fully, confirming seed viability. Addition of 50 mM NaCl did not significantly affect germination of either WT or *phyb-9* (Fig. 1A). However, under 125 mM NaCl, WT germination reached ~35% in Dark and ~75% in Light. In contrast, *phyb-9* germinated at only ~35% in Light, comparable to WT in Dark, and at ~20% in Dark. The modest light response in *phyb-9* likely reflects compensation by other photoreceptors (e.g., phyA). Similar patterns were observed for mannitol treatment (Fig. 1B). These results demonstrate that phyB is required for light-promoted

germination under salt and osmotic stress.

(2) To precisely assess phyB's role, we employed a standard phyB-dependent seed germination assay under stress. For phyB-OFF, seeds received 5 min far-red light to inactivate phyB; for phyB-ON, seeds received 5 min far-red followed immediately by 5 min red light to activate phyB. Seeds were then incubated in darkness. As shown in Fig. S1A, under phyB-OFF conditions, WT seeds did not germinate even on control medium. Under phyB-ON, WT germination was >90% on control medium and declined to ~40% (50 mM NaCl) and ~20% (125 mM NaCl). The *phyb-9* mutant failed to germinate under both phyB-ON and phyB-OFF conditions, similar to WT under phyB-OFF condition. Similar patterns were observed for mannitol treatment (Fig. S1B). These data provide clear evidence that phyB is essential for light-promoted tolerance to salt and osmotic stress during seed germination.

2. In lines 109–110, the authors state that pif3, pif4, and pif5 mutants are "indistinguishable from WT across all NaCl concentrations tested." Based on Figure 1C, there still appears to be a modest difference between WT and the pif4 mutant. This sentence should be rephrased to more accurately reflect the data.

Our Reply:

We agree with the reviewer and have revised the sentence as: "the *pif4* mutant showed a modest difference in germination, whereas *pif3* and *pif5* were indistinguishable from WT across the NaCl concentrations tested."

3. In Figure 2B, CML expression was analyzed using mature seeds, whereas the relevant phenotype concerns seed germination. If possible, it would be more appropriate to examine CML expression in imbibed seeds during germination to better correlate gene expression with the observed phenotypes.

Our Reply:

We thank the reviewer for this suggestion. To better correlate *CML20* expression with the germination process, we have now analyzed the transcript levels of *CML19*, *CML20*, *CML21* and *CML22* in dry and imbibed seeds. The results showed that the transcript level of *CML20* was substantially higher than those of its paralogs *CML19*, *CML21*, and *CML22* under all conditions examined. The abundant expression of *CML20* is consistent with its functional role in regulating seed germination. The updated expression data has been included in revised Fig. 2B.

4. The authors show interactions between CML20 with PIF1 and also phyB. Are these interactions affected under salt/osmotic conditions?

Our Reply:

We are grateful to the reviewer for this valuable suggestion. In our original manuscript, we demonstrated by *in vitro* pull-down assays that the CML20-PIF1 interaction is Ca^{2+}-dependent, whereas the CML20-phyB interaction is Ca^{2+}-independent. These findings suggest that the CML20-PIF1 interaction, rather than the CML20-phyB interaction, is likely modulated by stress-induced Ca^{2+} signals. Therefore, we focused our subsequent *in vivo* validation on the CML20-PIF1 module. To examine whether stress conditions affect the CML20-PIF1 interaction *in vivo*, we first monitored cytosolic Ca^{2+} dynamics using transgenic plants expressing the Ca^{2+}-sensitive luminescent reporter aequorin (AEQ). AEQ-based imaging revealed that NaCl or mannitol treatment rapidly triggered a significant elevation of cytosolic Ca^{2+} in imbibed seeds (Fig. 4E, 4F), confirming that salt and osmotic stress generate Ca^{2+} signals during seed imbibition. We then performed co-immunoprecipitation (Co-IP) assays using protein extracts from seeds imbibed under control conditions or with NaCl/ mannitol treatment. The results show that NaCl or mannitol treatment notably increased the amount of PIF1-Myc co-precipitated with CML20-YFP compared to control conditions (Fig. 4G, 4H), providing direct *in vivo* evidence that salt and osmotic stress enhance the CML20-PIF1 interaction.

5. In the proposed model, CML20 appears to bind Ca^{2+} under salt/osmotic stress and subsequently function in the nucleus, conceptually resembling the dynamic behavior of phyB. It would be helpful if the authors could clarify whether CML20 undergoes any subcellular localization changes between normal and salt/osmotic stress conditions, either experimentally or through additional discussion.

Our Reply:

We thank the reviewer for this insightful suggestion. In our study, we have demonstrated that salt and osmotic stress trigger Ca^{2+} signals in imbibed seeds, and that Ca^{2+} binding induces a conformational switch in CML20 from a closed to an open state. This structural change enhances the CML20-PIF1 interaction, which in turn modulates PIF1 self-association and DNA-binding activity, thereby transducing the stress signal. We agree with the reviewer that, in addition to this Ca^{2+}-dependent conformational regulation, other mechanisms, such as subcellular localization, may also be involved in the regulation of CML20 function. Although CaM/CML proteins are relatively small and may diffuse passively between the cytoplasm and nucleus, specific post-translational modifications (e.g., prenylation) have been shown to influence the localization of certain family members. In the revised manuscript, we have expanded the Discussion to explicitly consider the potential for stress-induced changes in CML20 localization and to highlight this as an important direction for future investigation.

6. Fig. 8A and B, last column shows stronger interaction. However, 8A appears to show two distinct bands and 8B shows a faster migrating band. Are these degradation products or cross-reacting bands migrating closely?

Our Reply:

We thank the reviewer for this careful observation. The faster-migrating bands below His-CML20 in the original Fig. 8A and 8B are likely degradation products resulting from protein freeze-thaw cycles. To address this issue, we repeated the pull-down assays with freshly purified proteins, and the additional bands were no longer present. The revised Fig. 8A and 8B now present the results from these new experiments. A note on the use of freshly purified proteins has been added to the Methods section.

7. Fig 2H/I and 8D/E, authors have performed epistasis analysis using a single concentration of salt/mannitol. It is better to perform using a concentration gradient to assess if phyB and pif1 are epistatic to cml20 under a broad range of stress condition.

Our Reply:

We thank the reviewer for this constructive suggestion. To address this point, we have now performed germination assays using a concentration gradient of NaCl and mannitol for the genotypes shown in Fig. 2H/I (Fig. 2G/H in the revised manuscript) and Fig. 8D/E.

For the epistasis analysis of Fig. 2H/I (Fig. 2G/H in the revised manuscript): At low stress (50 mM NaCl or 100 mM mannitol), Col-0, *pif1*, and *cml20 pif1* germinated at rates comparable to the unstressed control, whereas *cml20* already showed significant hypersensitivity. As stress increased (125–175 mM NaCl or 200–400 mM mannitol), germination of all genotypes declined progressively. Across the entire concentration range, *pif1* consistently exhibited hyposensitivity (higher germination than WT), *cml20* showed hypersensitivity (lower germination), and the *cml20 pif1* double mutant phenocopied *pif1* (Fig. S5). These results confirm that *PIF1* is epistatic to *CML20* over a broad range of salt and osmotic stress concentrations.

For the epistasis analysis of Fig. 8D/E: Across the tested NaCl or mannitol concentration gradients, *cml20*, *phyb-9*, and the *cml20 phyb-9* double mutant all displayed similarly enhanced hypersensitivity compared to Col-0, with no additive effect in the double mutant (Fig. S7). This supports the conclusion that phyB and CML20 function in the same genetic pathway under a broad range of stress conditions.

The new data have been included as Fig. S5 and S7 and are described in the revised Results section.

8. It is not clear what light condition they are using when assaying for salt/osmotic effects on seed germination. Please clarify in the figure legends.

Our Reply:

We have now clarified the light conditions in all relevant figure legends. The corresponding legends have been updated accordingly.

Reviewer #2:

I co-reviewed this manuscript with one of the reviewers who provided the listed reports. This is part of the Nature Communications initiative to facilitate training in peer review and to provide appropriate recognition for Early Career Researchers who co-review manuscripts.

Our Reply:

We thank Reviewer #2 for the time and effort in co-reviewing our manuscript. We also appreciate the contributions of the Early Career Researcher and support *Nature Communications*' initiative to provide peer-review training.

Reviewer #3:

This study identified the calmodulin-like protein CML20 as a key molecular node connecting light signaling (phyB-PIF1) with abiotic stress signaling (Ca^{2+}), revealing a new mechanism through which the inhibition of seed germination by PIF1 is rapidly relieved in a non-proteolytic manner under stress conditions. This discovery holds significant theoretical importance for understanding how plants balance germination timing in unfavorable environments and also demonstrates potential agricultural application value. However, several issues still need to be addressed before publication.

1. This study starts from the phenomenon of the phyB-PIF1 module responding to stress to regulate seed germination and directly transitions to Ca^{2+} signaling. It lacks in planta evidence showing that salt/osmotic stress can indeed induce a cytosolic Ca^{2+} increase sufficient to activate CML20 during the early stage of seed imbibition.

Our Reply:

We thank the reviewer for raising this important point. To directly determine whether salt or osmotic stress induces a cytosolic Ca^{2+} increase during seed imbibition, we performed *in planta* Ca^{2+} imaging using transgenic Arabidopsis expressing the Ca^{2+}-sensitive luminescent reporter aequorin (AEQ). Our results demonstrate that treatment with NaCl or mannitol rapidly triggered a significant elevation of cytosolic Ca^{2+} in imbibed seeds (Fig. 4E, 4F). These data provide direct evidence that salt and osmotic stress elicit Ca^{2+} signals at this critical stage. Together with our finding that the CML20-PIF1 interaction is Ca^{2+}-dependent, these results support the physiological relevance of stress-induced Ca^{2+} signaling in activating the CML20-PIF1 module during seed germination.

2. This study indicates that the interaction between CML20 and PIF1 is Ca^{2+}-dependent (Fig. 4) and that CML20 is induced by stress (Figs. 2C-D), proposing that stress induces Ca^{2+} to promote the CML20-PIF1 interaction. However, evidence demonstrating the effect of stress on the CML20-PIF1 interaction is lacking.

Our Reply:

We thank the reviewer for this constructive comment. As suggested by the reviewer, we have now shown that salt or osmotic stress induces a rapid increase in cytosolic Ca^{2+} during seed imbibition (Fig. 4E, 4F). To directly assess whether stress conditions enhance the CML20-PIF1 interaction *in vivo*, we performed Co-IP assays using protein extracts from seeds imbibed under control conditions or treated with NaCl/mannitol. The results show that NaCl or mannitol treatment notably increased the amount of PIF1-Myc co-precipitated with CML20-YFP compared to control conditions (Fig. 4G, 4H). These data provide direct *in vivo* evidence that salt and osmotic stresses strengthen the CML20-PIF1 interaction, thereby linking stress-induced Ca^{2+} signals to the regulation of this key module.

3. This study states that CML20 does not affect PIF1 protein abundance, but it remains unclear whether PIF1 protein abundance is affected under stress conditions. This point is crucial for an accurate description of the model in fig. 9.

Our Reply:

We thank the reviewer for this insightful suggestion. To determine whether CML20 affects PIF1 protein abundance under stress conditions, we have examined PIF1 protein levels in PIF1ox and PIF1ox/*cml20* seeds imbibed under control, NaCl- or mannitol-treated conditions. Immunoblot analysis showed that PIF1-Myc protein levels were similar between PIF1ox and PIF1ox/*cml20* under both control and stress conditions (Fig. S6). These results demonstrate that CML20 does not regulate PIF1 protein stability, even when seeds are exposed to salt or osmotic stress.

4. Figure 8 shows that phyB-Pfr can promote the CML20-PIF1 interaction, but the binding of phyB itself to CML20 is independent of light or Ca^{2+} (Fig. 7). This finding is interesting, but a mechanistic explanation is needed: Does phyB increase the encounter frequency between CML20 and PIF1, or does it induce a conformational change in PIF1 making it more recognizable by CML20? Some discussion on this is necessary.

Our Reply:

We thank the reviewer for this insightful suggestion. In the revised manuscript, we have extended the Discussion addressing the potential mechanisms by which phyB promotes the CML20-PIF1 interaction. We propose that phyB may serve as a scaffold that brings CML20 and PIF1 into closer proximity, thereby increasing their encounter frequency. Additionally, we cannot exclude the possibility that phyB exerts allosteric effects on PIF1 or CML20, altering their conformations and enhancing mutual recognition. These mechanistic possibilities represent interesting directions for future structural and biophysical studies.

5. Furthermore, as illustrated in the model, light-activated PHYB leads to the degradation of PIF1 protein. The authors claim that PHYB promotes the CML20-PIF1 interaction. It is required to elucidate how PHYB promotes the CML20-PIF1 protein interaction while simultaneously promoting PIF1 degradation.

Our Reply:

We thank the reviewer for raising this important mechanistic question regarding how phyB can promote both PIF1 degradation and the CML20-PIF1 interaction. We propose that these two regulatory modes operate in a context-dependent manner to fine-tune seed germination under varying environmental conditions.

In darkness, PIF1 protein accumulates to high levels and acts as a master repressor of germination. The transition from dark to light triggers rapid, phyB-mediated degradation of PIF1, which serves as an essential "on-switch" to relieve repression and initiate germination. Once seeds are exposed to continuous light, PIF1 levels are substantially reduced but maintain a low, steady-state. These residual PIF1 proteins retain repressive activity, which becomes particularly relevant when seeds encounter abiotic stress (e.g., salt or osmotic stress). Under these conditions, phyB associates with both CML20 and PIF1 and facilitates their interaction, thereby inhibiting PIF1's DNA-binding and transcriptional activity. Thus, the phyB-CML20-PIF1 module provides a reversible mechanism to modulate the activity of the remaining PIF1 pool, allowing seeds to maintain germination competence under stress conditions.

It is noteworthy that the interaction between phyB and PIF1 can lead to multiple regulatory outcomes. The specific mode of regulation, whether degradation or activity modulation, likely depends on the presence of distinct cofactors. For instance, the recruitment of specific kinases and E3 ligases is required for PIF1 degradation, whereas our study reveals that under stress conditions, Ca^{2+}-activated CML20 associates with the phyB-PIF1 complex and switches the regulatory output toward inhibiting PIF1 activity.

In summary, phyB-mediated PIF1 degradation primarily functions during the dark-to-light transition to trigger germination, whereas the phyB-facilitated CML20-PIF1 interaction operates under sustained light to integrate stress signals and fine-tune PIF1 activity. This two-tiered regulation enables plants to dynamically adjust germination in response to both light and stress cues. We have incorporated this discussion into the revised manuscript.

6. The in vitro pull-down assay used 0.5 mM $CaCl_2$ (Methods section), which is far higher than physiological cytosolic calcium peaks (typically 0.1-10 μM). Please explain the rationale for this concentration, or supplement with dose-response experiments within the physiological concentration range to verify physiological relevance.

Our Reply:

We thank the reviewer for raising this important point. We agree that the 0.5 mM $CaCl_2$ used in our *in vitro* assays exceeds typical physiological cytosolic Ca^{2+} levels. This concentration follows previous biochemical practices for studying Ca^{2+}-binding proteins, where 0.5–1 mM $CaCl_2$ is commonly used to ensure saturation of EF-hand motifs and to robustly detect Ca^{2+}-dependent interactions *in vitro* (Plotnikova et al., 2010; Shen et al., 2005; Zhao et al., 2023). We emphasize, however, that the physiological relevance of the Ca^{2+}-dependent CML20-PIF1 interaction is important and validated by our *in vivo* data: 1) NaCl or mannitol treatment rapidly elevates cytosolic Ca^{2+} in imbibed seeds (Fig. 4E, 4F), and 2) these same stress conditions significantly enhance the CML20-PIF1 interaction in co-IP assays performed using imbibed seeds (Fig. 4G, 4H). Thus, while the *in vitro* assay serves as a biochemical tool to characterize protein-binding capacity, the *in vivo* evidence links stress-induced Ca^{2+} signals to the potentiation of the CML20-PIF1 module under physiological conditions.

7.The Arabidopsis phyB-9 mutant carries an additional mutation in the VENOSA4 gene (Plant Physiol. 2018 Sep;178(1):3-6). Therefore, to confirm that the observed germination phenotype under stress conditions is specifically attributable to phyB loss of function, the authors should include other phyB mutant alleles in their analysis.

Our Reply:

We thank the reviewer for raising this point. We are aware of the report (Yoshida et al., 2018) indicating that some *phyb-9* lines carry a mutation in the *VENOSA4* gene. Since then, we have backcrossed our *phyb-9* line with Col-0 and genotyped the progeny. The *phyb-9* line used in our study therefore carries the wild-type *VENOSA4* gene. We have updated the “Plant Materials” section to specify the seed source and genetic status of this line and have cited the relevant report for clarity.

Reviewer #4:

This manuscript presents a comprehensive study addressing how light and calcium signaling pathways are integrated to regulate seed germination under abiotic stress conditions. The authors identify CALMODULIN-LIKE 20 (CML20) as a key calcium sensor that promotes germination tolerance to salt and osmotic stress, and demonstrate that CML20 directly interacts with the transcription factor PIF1 in a Ca^{2+}-dependent manner, thereby suppressing PIF1 self-association and DNA-binding activity through a non-proteolytic mechanism. Furthermore, the study shows that phytochrome B (phyB) independently associates with both CML20 and PIF1, facilitating the formation of a CML20–PIF1 complex and further attenuating PIF1 transcriptional activity. Genetic analyses place PIF1 downstream of CML20 and indicate that CML20 and phyB function within the same signaling pathway. Importantly, the work integrates a broad range of genetic, molecular, biochemical, and physiological approaches, which together provide strong and coherent support for the proposed phyB–CML20–PIF1 signaling

module.

Major comments

A central pillar of the study relies on the phenotypic and molecular characterization of the cml20 mutant derived from the T-DNA insertion line SALK_079974. While the data obtained using this line are internally consistent, conclusions drawn from a single mutant allele may be confounded by allele-specific effects, secondary mutations, or positional effects associated with T-DNA insertions. Therefore, independent validation using additional cml20 alleles—such as other insertion lines or CRISPR-generated loss-of-function mutants—is essential to robustly attribute the observed phenotypes to CML20 function and to strengthen the generality and reliability of the proposed model.

Our Reply:

We agree that analysis using multiple independent alleles is valuable for genetic validation. While we do not currently have an additional *cml20* allele available to perform such experiments within the revision period, we would like to respectfully emphasize that genetic complementation is a widely accepted and rigorous alternative approach to establish gene-phenotype causality. In this study, we introduced a Myc-tagged full-length CML20 construct into the *cml20* mutant background. As shown in Fig. 2E, 2F, S4B and S4C, two independent transgenic lines fully rescued the stress-sensitive germination phenotype of the *cml20* mutant. These results demonstrate that the observed defects are specifically attributable to loss of CML20 function, rather than to off-target effects or secondary mutations associated with the T-DNA insertion.

A major concern regarding the germination assays is the use of exogenous sucrose, which is known to promote seed germination by providing an external carbon source and thereby alleviating the requirement for early establishment of photosynthetic activity. As a result, sucrose can partially bypass developmental and energetic constraints that are normally imposed during germination under stress conditions, including salinity and altered light regimes. In addition to its well-documented role as a signaling molecule, sucrose therefore fundamentally alters the physiological context of germination, making it difficult to attribute the observed phenotypes specifically to salt stress or light–phytochrome signaling. For this reason, germination assays aimed at dissecting salt and light responses are typically performed in the absence of sucrose, often on reduced-strength (½×) MS medium to minimize additional osmotic or nutritional effects. The authors should justify the inclusion of sucrose or confirm that the key conclusions hold under sugar-free conditions.

Our Reply:

We agree with the reviewer that it is important to verify our key findings under conditions that minimize potential effects from sucrose and additional osmotic or nutritional factors. To address this concern, we re-evaluated the germination phenotypes of all relevant genotypes (including those presented in Fig. 1D, 1E, 2E, 2F,

2G, 2H, 8D, and 8E) under three different media: MS medium with 1% sucrose (as used originally), ½ MS medium with 1% sucrose, and ½ MS medium without sucrose.

Although the absolute germination rates slightly varied among the three media, the relative phenotypic differences between genotypes remained fully consistent across all conditions. Specifically, we observed that all seeds germinated fully under non-stress conditions, and the same phenotypic patterns were maintained across the three different media with stress treatment: (1) *pif1* mutants showed higher germination rates than WT, while *35S*:*PIF1-Myc*/*pif1* lines showed lower germination; (2) *cml20* mutants exhibited reduced germination, whereas *35S*:*CML20-Myc/cml20* lines displayed enhanced germination; (3) the *cml20 pif1* double mutant phenocopied *pif1*; and (4) the *cml20 phyb-9* double mutant behaved similarly to each single mutant, exhibiting hypersensitivity to stress.

These results, now provided in Fig. S8 and S9, confirm that our key conclusions are robust and are not altered by the presence or absence of sucrose in the medium.

The study assumes that salt/osmotic stress generates a Ca^{2+} signal relevant to CML20; however, no direct measurement of Ca^{2+} dynamics in seeds is provided. It is recommended to include, at minimum, an explicit discussion of this point, or a reference to relevant work in seeds. Alternatively, an experiment using a Ca^{2+} chelator (e.g., EGTA or BAPTA) during germination could strengthen the functional link between stress, Ca^{2+} signaling, and CML20 activity.

Our Reply:

We thank the reviewer for this insightful suggestion. To directly assess whether salt or osmotic stress elicits cytosolic Ca^{2+} increases in imbibed seeds, we performed live Ca^{2+} imaging using transgenic Arabidopsis expressing the aequorin (AEQ) Ca^{2+} reporter. Our results show that treatment with NaCl or mannitol rapidly induced a pronounced elevation in cytosolic Ca^{2+} levels in imbibed seeds (Fig. 4E, 4F). These data provide direct evidence that such stresses trigger a Ca^{2+} signal, thereby strengthening the functional link between stress-induced Ca^{2+} signaling and CML20 activity.

Minor comment:

For clarity and consistency, it would be preferable to label the y-axis as “Relative transcript level” (e.g., Relative CML20 transcript level), which more accurately reflects the normalized RT-qPCR-based expression data.

Our Reply:

We thank the reviewer for this suggestion. Accordingly, we have updated the y-axis labels in all RT-qPCR figures (Fig. 2B–2D, S2B and S4A) to clearly reflect the normalized expression data.

We thank all reviewers again for their constructive feedback. We believe that the additional experiments and revisions have substantially strengthened the manuscript.

Reference

Ma, L., Han, R., Yang, Y.Q., Liu, X.N., Li, H., Zhao, X.Y., Li, J.F., Fu, H.Q., Huo, Y.D., Sun, L.P., et al. (2023). Phytochromes enhance SOS2-mediated PIF1 and PIF3 phosphorylation and degradation to promote Arabidopsis salt tolerance. Plant Cell 35, 2997-3020.

Plotnikova, O.V., Pugacheva, E.N., Dunbrack, R.L., and Golemis, E.A. (2010). Rapid calcium-dependent activation of Aurora-A kinase. Nature Communications 1061.

Ren, Z.H., Zheng, Z.M., Chinnusamy, V., Zhu, J.H., Cui, X.P., Iida, K., and Zhu, J.K. (2010). RAS1, a quantitative trait locus for salt tolerance and ABA sensitivity in *Arabidopsis*. PNAS 107, 5669-5674.

Shen, X.C., Valencia, C.A., Szostak, J.W., Dong, B., and Liu, R.H. (2005). Scanning the human proteome for calmodulin-binding proteins. PNAS 102, 9734-9734.

Yoshida, Y., Sarmiento-Mañús, R., Yamori, W., Ponce, M.R., Micol, J.L., and Tsukaya, H. (2018). The Arabidopsis *phyB-9* mutant has a second-site mutation in the *VENOSA4* gene that alters chloroplast size, photosynthetic traits, and leaf growth. Plant Physiology 178, 3-6.

Yu, L.H., Wu, J., Zhang, Z.S., Miao, Z.Q., Zhao, P.X., Wang, Z., and Xiang, C.B. (2017). *Arabidopsis* MADS-Box transcription factor AGL21 acts as environmental surveillance of seed germination by regulating expression. Molecular Plant 10, 834-845.

Zhao, Y., Shi, H., Pan, Y., Lyu, M., Yang, Z.X., Kou, X.X., Deng, X.W., and Zhong, S.W. (2023). Sensory circuitry controls cytosolic calcium-mediated phytochrome B phototransduction. Cell 186, 1230-1243.

MS ID#: NCOMMS-25-93787A

MS TITLE: CML20 integrates phytochrome and calcium signaling pathways to optimize seed germination under abiotic stress

Reviewer #1:

The authors have adequately addressed most of my concerns except they did not examine the subcellular localization of CML20. They have CML20-GFP transgenic plants in PIF1OX background. This can be done easily to examine where the protein is located before and after stress conditions. This can be very helpful in the context of this story.

Our Reply:

We thank the reviewer for this valuable suggestion. In response, we performed nuclear-cytoplasmic fractionation followed by Western blotting using *35S:CML20-YFP/cml20* and *35S:CML20-Myc/cml20* transgenic seeds. Imbibed seeds were treated with NaCl or mannitol, and protein fractions were probed with anti-GFP or anti-Myc antibodies. Both CML20-YFP and CML20-Myc were detected in the nuclear and cytoplasmic fractions under all conditions, and stress treatment did not cause any obvious redistribution relative to the untreated control. These results demonstrate that CML20 does not undergo stress-induced subcellular relocalization. We have included these data as Figure S7 in the revised manuscript.

Reviewer #2:

I co-reviewed this manuscript with one of the reviewers who provided the listed reports. This is part of the Nature Communications initiative to facilitate training in peer review and to provide appropriate recognition for Early Career Researchers who co-review manuscripts.

Our Reply:

We thank Reviewer #2 for the time and effort in co-reviewing our manuscript. We also appreciate the contributions of the Early Career Researcher and support *Nature Communications*' initiative to provide peer-review training.

Reviewer #3:

The author has fully addressed my concerns in the revised version. I have no further worries, and I believe the current version is suitable for publication.

Our Reply:

We sincerely thank the reviewer for the positive evaluation and are pleased that our revisions have fully addressed all concerns.

Reviewer #4:

I would like to commend the authors for their thorough and thoughtful responses to the reviewers' comments; their efforts to address the feedback and improve the manuscript are highly appreciated. There is, however, just one remaining point that requires further attention before publication.

The authors' effort to provide a complementation line for the cml20 mutant is highly valued and indeed confirms that the observed phenotype is linked to the functional loss of this locus. However, relying on a single T-DNA insertion line remains a concern.

While complementation rescues the phenotype, it cannot rule out allele-specific artifacts. For instance, depending on the exact insertion site, a single T-DNA mutant can sometimes produce truncated transcripts with dominant-negative effects, or cause unintended neighboring gene disruption (position effects) that could be coincidentally altered during tissue culture or transformation. Furthermore, passenger mutations linked to this specific line cannot be entirely excluded.

Characterizing two independent mutant alleles is the standard benchmark in plant genetics to robustly confirm a causal link between a gene and a phenotype. Therefore, while a full characterization might not be necessary given that the complementation is clean, the authors should at least provide basic phenotypic data for a second independent cml20 allele to conclusively solidify their findings.

Our Reply:

We thank the reviewer for the detailed explanation regarding the importance of a second independent allele. In response, we have used an additional T-DNA insertion line for *CML20* (CS925331), designated *cml20-2* (Fig. S4A). RT-qPCR showed that *CML20* transcript levels are markedly reduced in both *cml20* and *cml20-2* mutants (Fig. S4B). Germination assays under salt and osmotic stress demonstrated that *cml20-2* exhibits hypersensitivity similar to that of the original *cml20* mutant (Fig. S4C). The consistent phenotype observed in two independent alleles supports that the stress-sensitive germination phenotype is caused by loss of *CML20* function, ruling out allele-specific artifacts. We thank the reviewer again for this constructive suggestion, which has strengthened the genetic confirmation of our findings.

MS ID#: NCOMMS-25-93787B

MS TITLE: CML20 integrates phytochrome and calcium signaling pathways to optimize seed germination under abiotic stress

REVIEWERS' COMMENTS

Our Reply:

We note that the reviewers have no further comments. We sincerely thank the reviewers for their positive evaluation and are pleased that our revisions have fully addressed all concerns.
